# Species-Specific Gene Expansion of the *Cellulose synthase* Gene Superfamily in the Orchidaceae Family and Functional Divergence of Mannan Synthesis-Related Genes in *Dendrobium officinale*

**DOI:** 10.3389/fpls.2022.777332

**Published:** 2022-06-03

**Authors:** Yunzhu Wang, Kunkun Zhao, Yue Chen, Qingzhen Wei, Xiaoyang Chen, Hongjian Wan, Chongbo Sun

**Affiliations:** ^1^Institute of Horticulture Research, Zhejiang Academy of Agricultural Sciences, Hangzhou, China; ^2^Institute of Vegetable Research, Zhejiang Academy of Agricultural Sciences, Hangzhou, China; ^3^Seed Management Terminal of Zhejiang, Hangzhou, China

**Keywords:** *Cellulose synthase*, *CesA/Csl*, Orchidaceae, gene expansion, polysaccharide synthesis

## Abstract

Plant *Cellulose synthase* genes constitute a supergene family that includes the *Cellulose synthase* (CesA) family and nine *Cellulose synthase*-like (Csl) families, the members of which are widely involved in the biosynthesis of cellulose and hemicellulose. However, little is known about the *Cellulose synthase* superfamily in the family Orchidaceae, one of the largest families of angiosperms. In the present study, we identified and systematically analyzed the *CesA/Csl* family members in three fully sequenced Orchidaceae species, i.e., *Dendrobium officinale*, *Phalaenopsis equestris*, and *Apostasia shenzhenica*. A total of 125 *Cellulose synthase* superfamily genes were identified in the three orchid species and classified into one CesA family and six Csl families: CslA, CslC, CslD, CslE, CslG, and CslH according to phylogenetic analysis involving nine representative plant species. We found species-specific expansion of certain gene families, such as the CslAs in *D. officinale* (19 members). The *CesA/Csl* families exhibited sequence divergence and conservation in terms of gene structure, phylogeny, and deduced protein sequence, indicating multiple origins via different evolutionary processes. The distribution of the *DofCesA/DofCsl* genes was investigated, and 14 tandemly duplicated genes were detected, implying that the expansion of *DofCesA/DofCsl* genes may have originated via gene duplication. Furthermore, the expression profiles of the *DofCesA/DofCsl* genes were investigated using transcriptome sequencing and quantitative Real-time PCR (qRT-PCR) analysis, which revealed functional divergence in different tissues and during different developmental stages of *D. officinale*. Three *DofCesAs* were highly expressed in the flower, whereas *DofCslD* and *DofCslC* family genes exhibited low expression levels in all tissues and at all developmental stages. The 19 *DofCslAs* were differentially expressed in the *D. officinale* stems at different developmental stages, among which six *DofCslAs* were expressed at low levels or not at all. Notably, two *DofCslAs* (*DofCslA14* and *DofCslA15*) showed significantly high expression in the stems of *D. officinale*, indicating a vital role in mannan synthesis. These results indicate the functional redundancy and specialization of *DofCslAs* with respect to polysaccharide accumulation. In conclusion, our results provide insights into the evolution, structure, and expression patterns of *CesA/Csl* genes and provide a foundation for further gene functional analysis in Orchidaceae and other plant species.

## Introduction

cellulose, hemicellulose, and pectin are three major types of polysaccharides in plant cell walls and play vital roles in controlling cell shape, expansion, overall development, and interactions with the environment (McFarlane et al., 2014). cellulose is a paracrystalline polymer of β-1,4 glucan chains and is the major determinant of the load-bearing capacity of cell walls constituting approximately one-third of the total plant mass (Nishiyama, 2009; McFarlane et al., 2014). Hemicelluloses are polysaccharides that have equatorial β-(1→4)-linked backbones, including xyloglucans, xylans, mannans, glucomannans, and β-(1→3,1→4)-glucans (Scheller and Ulvskov, 2010). Hemicellulose and pectin polysaccharide matrix fill in the gaps between cellulose microfibrils.

The *Cellulose synthase* (CesA) superfamily comprises the CesA family and nine CesA-like (Csl) families, which all belong to the glycosyltransferase-2 (GT2) superfamily, typically with a catalytic domain containing a DDDQXXRW motif (Pear et al., 1996; Richmond and Somerville, 2000). The number of *CesA* genes is rather stable among plant species, usually around ten members; however, these genes underwent expansion, which then had 18 genes (Yin et al., 2009). Csl family members also underwent large gene expansion, from the presence of a single CslA/C-like gene in green algae to up to 50 genes in terrestrial plant species. The expansion of plant *Cellulose synthase* genes is closely linked to major events in the evolution of plant and algal lineages, including multicellularity, terrestrialization, and vascularization (Popper et al., 2011). cellulose is synthesized by plasma membrane-localized *Cellulose synthase* complexes (CSCs) with access to the GDP-glucose pool in the cytosol. In plants, the first *CesA* gene in cotton (*Gossypium hirsutum*) fibers was identified based on the sequence similarity to a bacterial *CesA* gene (Pear et al., 1996). To date, *CesA/Csl* family genes have been identified in many plant species, including *Arabidopsis* (Persson et al., 2007), tomato (Song et al., 2018), rice (Shu et al., 2007), sorghum (Paterson et al., 2009), *Populus trichocarpa* (Takata and Taniguchi, 2015), *Pyrus bretschneideri* (Li et al., 2020), and cotton (Li et al., 2017; Cui et al., 2020). Ten *CesA* genes are present in the *Arabidopsis* genome and are required for primary (*CesA1−3*, *−5*, *−6*, and *−9*) and secondary (*CesA4*, *−7*, and *−8*) cell wall synthesis (Taylor et al., 2003; Takata and Taniguchi, 2015). Mutations of *cesa1* and *cesa3* are gamete lethal, whereas single-knockout mutants of the other primary cell wall-related *CesA* genes exhibit more moderate phenotypes (Desprez et al., 2007; Persson et al., 2007).

Hemicellulose is synthesized in the Golgi apparatus by membrane-localized enzymes encoded by *Cellulose synthase-like* (*Csl*) genes, which share relatively high sequence similarity to those of *CesA* family genes. In plants, the Csl families usually comprise 30–50 members, which are classified into nine subgroups: CslA-CslH and CslJ (Keegstra and Walton, 2006; Fincher, 2009; Yin et al., 2014). Of these, CslA, CslC, and CslD are commonly found in all land plants, and CslE and CslJ are common to angiosperms (Yin et al., 2009, 2014; Popper et al., 2011). CslH and CslF are specific to grasses, whereas CslB and CslG are found in non-grass angiosperms (Keegstra and Walton, 2006; Suzuki et al., 2006; Popper et al., 2011; Yin et al., 2014). *Csl* family genes are reported to be involved in the synthesis of various cell wall polysaccharides (Kaur et al., 2017). *CslAs* encode proteins with both mannan and glucomannan synthase activity (Liepman et al., 2010). In *Arabidopsis*, the stems of *csla*-knockout mutants have no glucomannan, indicating that *CslA* family genes have an exclusive role in mannan biosynthesis (Goubet et al., 2009). The members of the *CslC* family are involved in the synthesis of the β-1,4-linked glucan backbone of xyloglucan in *Tropaeolum majus* (Cocuron et al., 2007). *CslDs* in *Arabidopsis* are involved in mannan and *Cellulose synthesis*, especially in tip-growing root hairs and pollen tubes (Park et al., 2011). In tobacco, *CslD-*overexpressing plants have high mannan synthase activity (Verhertbruggen et al., 2011). The rice *OsCSLD1* gene is required for root hair morphogenesis (Kim et al., 2007). The *CslF*, *CslH*, and *CslJ* genes are involved in the synthesis of mixed-linkage glucan polymers in grasses such as rice and barley (Burton et al., 2006; Doblin et al., 2009; Fincher, 2009). However, the roles of the remaining *Csl* family members (*CslB*, *CslD*, *CslE*, *CslG*) remain unclear.

Orchidaceae is one of the largest families of angiosperms whose members exhibit vastly different morphotypes, lifestyles, and remarkable adaptations to environmental conditions. *Dendrobium* is one of the largest genera of the Orchidaceae family, containing approximately 1,450 species (Zhang et al., 2016); these species are characterized by bioactive ingredients with immunomodulatory hepatoprotective activities, such as dendrobine and polysaccharides (Ng et al., 2012). *Dendrobium officinale* contains abundant polysaccharides in flesh stems, primarily glucomannan (GM) and galactoglucomannan (GGM) (Xing et al., 2014). Previous studies have shown that *Csl* genes, especially *CslAs*, play important roles in the synthesis of GM and GGM. However, little is known about the evolutionary pattern and functional diversification of the *Csl* genes involved in polysaccharide content accumulation. In this study, we performed a global analysis of the identification and characterization of the *CesA/Csl* family in three fully sequenced orchid species: *D. officinale* (Zhang et al., 2016), *P. equestris* (Cai et al., 2014), and *A. shenzhenica* (Zhang et al., 2017). Moreover, the contents of water-soluble polysaccharides and constituting monosaccharides were also measured to elucidate the potential functional divergence of the *Csl* genes in *D. officinale*. The results provide insights into the evolutionary patterns and functional divergence of *CesA/Csls* in Orchidaceae species.

## Materials and Methods

### Identification and Characterization of *Cellulose synthase*s in the Orchidaceae Family

The genome sequences of three Orchidaceae species, *D. officinale* (Zhang et al., 2016), *P. equestris* (Cai et al., 2014), and *A. shenzhenica* (Zhang et al., 2017) were downloaded from the NCBI database^1^. The *Cellulose synthase* family proteins contain two different pfam domains: PF03552 for CesAs and PF00535 for Csls. The seed sequences of the two domains were then downloaded from the TIGRFAMs database^2^. HMMsearch from the HMMER suite (version 3.1; Finn et al., 2011) was used to search for *Cellulose synthase* proteins in *D. officinale*, *P. equestris*, and *A. shenzhenica* with a cutoff *E*-value of 1e^––4^. The molecular weight and theoretical isoelectric point (pI) of the proteins were predicted using the ExPASy website^3^. Transmembrane domains and subcellular locations were predicted using TMHMM^4^ and WoLF PSORT^5^, respectively.

### Phylogenetic Analysis of the *CesA/Csl* Proteins

The amino acid sequences of *CesA/Csls* were retrieved for nine species, including algae, mosses, lycophytes, monocots, and dicots: *Chlamydomonas reinhardtii* (*Cre*), *Volvox carteri* (*Vca*), *Physcomitrella patens* (*Ppa*), *Selaginella moellendorffii* (*Smo*), *Oryza sativa* (*Osa*), *A. shenzhenica* (*As*), *D. officinale* (*Dof*), *P. equestris* (*Peq*), and *Arabidopsis thaliana* (*AT*). The protein sequences were downloaded from the Pfam database^6^. MEGA 6 (Tokyo Metropolitan University, Tokyo, Japan; Tamura et al., 2013) was used to analyze the protein sequences and construct phylogenetic trees. First, the MUSCLE program of MEGA 6 was used for multiple sequence alignment, and then the maximum likelihood method (ML) method with the Jones--Taylor--Thornton (JTT) model was used to construct a phylogenetic tree with 1,000 bootstrap replicates. A partial deletion with a site coverage cutoff of 70% was used for gap treatment. The phylogenetic trees were visualized and modified using MEGA 6 and iTOL^7^.

### Gene Structure, Distribution, and Protein Sequence Analyses

The Gene Structure Display Server tool^8^ (v2.0; Hu et al., 2015) was used to analyze the gene structure of all the *CesA/Csls* identified in the three Orchidaceae species. Gene distribution in the genome of *D. officinale* was visualized using the TBtools program (Chen et al., 2020), and conserved domains were identified with the NCBI Web CD-Search tool^9^. MEME software^10^ (v4.11.0) was used to search for sequences of motifs of *CesA/Csl* proteins, with a motif window length from 10 to 100 bp (Bailey et al., 2009), with the maximum number of motifs set at 10, and where motifs present in at least three proteins were considered true motifs. Multiple alignments of *CesA/Csls* in the three orchid species were conducted using DNAMAN software (version 9; Lynnon Biosoft Company, Quebec, QC, Canada).

### Expression Analysis of *CesA/Csl* Genes in Different Organs and at Different Developmental Stages of *Dendrobium officinale*

Two transcriptome sequencing analyses were performed using four different organs of a 3-year-old *D. officinale* and the stems of three developmental stages of *D. officinale*. The four different organs of the 3-year-old *D. officinale* used in the present study include flower, leaf, flower, and root. Three different *D. officinale* developmental stages were investigated: 1-year old (Y1), 2 years old (Y2), and 3 years old (Y3). The plants were grown in glasshouses at the Mulberry Field Station of Zhejiang Academy of Agriculture Science (Hangzhou, China). The tissues were collected and frozen in liquid nitrogen and stored at –80°C until use. For each tissue, five plants were treated as an independent biological replicate, and three biological replicates were performed. Total RNA was extracted using TRIzol reagent (Invitrogen, Carlsbad, CA, United States) according to the manufacturer’s instructions and sequenced on an Illumina HiSeq 2000 platform. Subsequently, the expression profiles of all *D. officinale* genes were obtained with FPKM (fragments per kilobase of exon per million fragments mapped) values using Cufflinks software^11^ (v2.2.1) according to annotated gene models with a GFF file. The expression profiles of the *CesA/Csl* genes from each sample were analyzed using the HemI program^12^ with the average hierarchical clustering method.

### Determination of Total Water-Soluble Polysaccharide Contents

Stems of *D. officinale* plants at five different growth stages, i.e., 3 months (3M), 9 months (9M), 1 year (Y1), 2 years (Y2), and 3 years (Y3), were collected (three replicates for each sample) and dried in an oven at 105°C until constant weight. The 3-M and 9-M *D. officinale* seedlings were cultured on half-strength Murashige and Skoog (MS) (Murashige and Skoog, 1962) media containing 0.1% activated carbon, 2% sucrose, and 0.6% agar (pH 5.4) in a growth chamber (25.5 ± 1°C, 45 μmol m-2 s-1 irradiance, 12-h light/dark photoperiod, 60% relative humidity). Y1, Y2, and Y3 *D. officinale* plants were grown in glasshouses at the Mulberry Field Station of Zhejiang Academy of Agriculture Science (Hangzhou, China). The samples were shattered into fine powder independently by a mixing mill (MM 400, Retsch). The total polysaccharide was extracted using the water extraction and alcohol precipitation method, and the contents of total polysaccharides were measured using the phenol–sulfuric acid method as described by Wang et al. (2021). Glucose was used as a reference for subsequent calculations in which total polysaccharides (μg/g dry weight) = (A + 0.0037)÷7.981 × V1÷V2 × V3÷W × 1000 = 626. 49 × (A + 0.0037)÷W, where V1 represents the redissolved volume after alcohol precipitation (1 mL), V2 represents the volume of alcohol precipitation (0.2 ml), V3 represents the volume of water added during extraction (1 mL), and W represents sample weight in grams (1,000 g), serving as a coefficient converting milligrams to micrograms.

### Determination of Monosaccharide Contents

After drying, the samples from the *D. officinale* stems of the five growth stages were shattered into fine powder independently by a mixing mill (MM 400, Retsch). The constituting monosaccharides, including mannose, glucose, and galactose, were extracted using the GC–MS/MS method (Sun et al., 2016). Briefly, 20 mg of powder was diluted in a 500 μL solution and the extracts were centrifuged at 14,000 rpm at 4°C for 3 min. Then, the supernatants were mixed, evaporated, and freeze-dried. The residue was used for further derivatization. The small-molecule carbohydrates and 100 μL of a solution of methoxyamine hydrochloride in pyridine (15 mg/mL) were mixed together and incubated at 37°C for 2 h. Then, 100 μL of BSTFA was added and the mixture was maintained at 37°C for 30 min after being vortexed. The mixture was subsequently diluted and analyzed by GC–MS/MS according to the methods of Gómez-González et al. (2010) and Sun et al. (2016) with modifications. Agilent 7890B gas chromatograph coupled to a 7000D mass spectrometer equipped with a DB-5MS column (30 m length × 0.25 mm i.d. × 0.25 μm film thickness, J&W Scientific, Santa Clara, CA, United States) was employed for GC–MS/MS analysis of the monosaccharides.

### RNA Extraction and Quantitative Real-Time PCR Analysis

Total RNA was extracted from all the samples of *D. officinale* stems at the five growth stages (3M, 9M, Y1, Y2, and Y3) using TRIzol reagent (Invitrogen, Carlsbad, CA, United States). The potential contaminating genomic DNA was eliminated with DNase I. The quality of the RNA samples was checked with a NanoDrop 2000 spectrophotometer (Thermo Fisher Scientific, Beijing, China) and 1% denaturing agarose gels. The RNA was used as a template for first-strand cDNA synthesis using PrimeScript reverse transcriptase (TaKaRa Biotechnology, Dalian, China). Gene-specific primers were designed with the Primer Premier 5.0 program. The *DnActin* (comp205612_c0) gene was used as an internal standard for normalizing the gene expression data. The *DofCesA/DofCsl* expression levels were analyzed using a quantitative Real-time PCR (qRT–PCR) assay, which was performed by using a SYBR Green qPCR Kit (TaKaRa Biotechnology, Dalian, China) and a Stratagene Mx3000P thermocycler (Agilent, Santa Clara, CA, United States). The PCR program was as follows: 95°C for 5 min followed by 40 cycles at 95°C for 15 s and then 60°C for 30 s. The relative gene expression levels were calculated with the 2^–ΔΔ*Ct*^ method (Livak and Schmittgen, 2001). Three biological replicates were subjected to the analysis, each with three technical replicates. The expression levels in the different tissues were visualized using GraphPad Prism (v. 8.4.3).

### Statistical Analyses

Statistical analysis was performed, and the average values and standard errors of the three replicates were calculated. SPSS software (v. 16.0) was used to determine the significant differences in polysaccharide and monosaccharide contents between the different developmental stages using a one-way ANOVA procedure and *post hoc* analysis. The value *p* < 0.05 indicates a significant difference and is represented by an asterisk (*) in the figures, and *p* < 0.01 indicates a very significant difference and is represented by two asterisks (^**^) in the figures. (***) indicates *p* value < 0.001 and (****) indicates *p* value < 0.0001.

## Results

### Genome-Wide Characterization of the *Cellulose synthase* Superfamily in Orchidaceae Species

**FIGURE 1:**
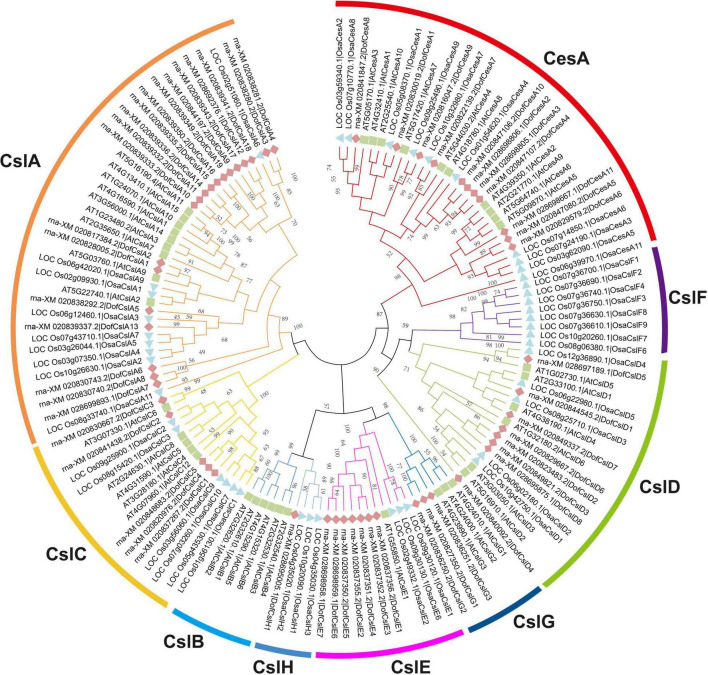
Phylogenetic tree of the *Cellulose synthase* superfamily in *D. officinale* (Dof), rice (Osa) and *Arabidopsis* (At). The phylogenetic tree was constructed using MEGA 6.0 with the neighbor-joining (NJ) method and 1,000 bootstrap replicates. The *CesA/Csl* proteins were grouped into one CesA family and eight Csl families: CesA, CslA, CslB, CslC, CslD, CslE, CslF, CslG, and CslH. The families are marked by different arc lines and branch colors, and individual species are distinguished by triangles, squares, or rhombuses in different colors.

To investigate the potential roles of *Cellulose synthase* superfamily genes in orchids, we performed a genome-wide identification and characterization of *CesA/Csl* genes in three sequenced Orchidaceae species: *D. officinale* (Zhang et al., 2016), *P. equestris* (Cai et al., 2014), and *A. shenzhenica* (Zhang et al., 2017). A total of 125 *Cellulose synthase* superfamily genes were identified in the three orchid species, which were designated ‘DofCesA/DofCsl’ for *D. officinale*, ‘PeqCesA/PeqCsl’ for *P. equestris*, and ‘AsCesA/AsCsl’ for *A. shenzhenica* (Supplementary Table S1). The CesA superfamily members in the Orchidaceae family were classified into seven families: CesA, CslA, CslC, CslD, CslE, CslG, and CslH according to the sequence similarities with that of *A. thaliana* and *O. sativa* (Figure 1, Supplementary Figure S1, and Supplementary Table S1).

We identified 54 *CesA/Csl* proteins from the *D. officinale* genome, which comprised 11 CesAs and 43 Csls (Table 1). The 54 proteins were further classified into DofCesA (11 members) and six different Csl families, i.e., DofCslA (19 proteins), DofCslC (5 proteins), DofCslD (8 proteins), DofCslE (7 proteins), DofCslG (3 proteins), and DofCslH1, according to the genome annotation information and sequence similarity with those of *Arabidopsis* and rice (Figure 1 and Table 1). *P. equestris* was identified with 37 *CesA/Csl* proteins, which were classified into PeqCesA (8 proteins) and six *Csl* families: PeqCslA (10 proteins), PeqCslD (10 proteins), PeqCslH (3 proteins), PeqCslE (4 proteins), PeqCslC1, and PeqCslG1. The *A. shenzhenica* genome was identified with 34 *CesA/Csl* proteins, including AsCesA (9 proteins), AsCslA (10 proteins), AsCslC (4 proteins), AsCslD (7 proteins), AsCslG (2 proteins), AsCslE1, and AsCslH1. These results indicated that the *Cellulose synthase* superfamily in *D. officinale* had greatly expanded, whereas that in the other two Orchidaceae species was similar to *Arabidopsis* (Persson et al., 2007), tomato (Song et al., 2018), and rice (Wang et al., 2010). Notably, although the number of DofCesAs was similar to that in other species, the DofCsl family had significantly expanded, especially DofCslA.

**TABLE 1:** Physical and molecular characteristics of *Cellulose synthase* superfamily genes in *D. officinale*.

Name	Gene_Protein_ID	Start	End	Exon	Intron	CDS length (bp)	Size (aa)	pI	MW	TM domains	Subcellular localization
DenCesA1	rna-XM_020830019.2	2543401	2553956	16	15	3261	1086	6.70	121975.30	8	plas
DenCesA2	rna-XM_028698806.1	345324	356661	10	9	2214	737	6.27	82859.64	6	plas, vacu
DenCesA3	rna-XM_028698805.1	345273	356662	10	9	2277	758	6.11	85463.52	6	plas
DenCesA4	rna-XM_020847107.2	345324	356662	11	10	2607	868	5.79	97737.54	6	plas
DenCesA5	rna-XM_020847080.2	345273	765345	14	13	3273	1090	6.92	122919.74	8	plas
DenCesA6	rna-XM_020829579.2	344955	221410	14	13	3276	1091	7.04	123193.16	8	plas
DenCesA7	rna-XM_020822139.2	758719	181251	11	10	2715	904	8.68	103177.75	8	plas
DenCesA8	rna-XM_020841847.2	214425	3169725	16	15	3222	1073	6.80	120382.50	6	plas
DenCesA9	rna-XM_020816047.2	171675	3037542	13	12	3246	1081	6.62	122499.99	8	plas, golg_plas
DenCesA10	rna-XM_020847106.2	344955	356662	13	12	2943	980	6.03	110590.23	6	plas, vacu, E.R.
DenCesA11	rna-XM_028698667.1	814718	820703	8	7	1626	541	5.63	61528.70	2	plas, golg_plas
DenCslA1	rna-XM_020828005.2	355167	374826	9	8	1587	528	8.98	60666.30	5	plas
DenCslA2	rna-XM_020817384.2	770742	779370	9	8	1611	536	9.08	61122.61	5	plas
DenCslA3	rna-XM_020838280.2	4890932	4895458	10	9	1680	559	9.13	64684.74	5	plas, E.R.
DenCslA4	rna-XM_020838281.2	4860583	4872379	10	9	1659	552	9.29	64699.91	5	plas
DenCslA5	rna-XM_020838292.2	4917570	4945582	9	8	1719	572	6.58	64724.43	5	plas
DenCslA6	rna-XM_020830743.2	476954	483146	9	8	1653	550	8.94	62803.26	5	plas, vacu, E.R.
DenCslA7	rna-XM_028699893.1	502377	515187	9	8	1551	516	8.70	59350.76	4	plas, E.R.
DenCslA8	rna-XM_020830740.2	546739	553430	9	8	1650	549	8.90	62969.11	5	plas
DenCslA9	rna-XM_020844197.2	193619	196422	9	8	1494	497	8.32	57409.47	5	plas, golg_plas
DenCslA10	rna-XM_020839333.2	682541	687971	9	8	1596	531	8.87	60970.18	5	plas, golg_plas
DenCslA11	rna-XM_020839332.2	682535	687971	10	9	1695	564	8.76	64714.50	5	plas
DenCslA12	rna-XM_028692376.1	743655	816186	9	8	1560	519	8.86	59431.54	5	plas, cyto_plas, E.R.
DenCslA13	rna-XM_020839337.2	656622	672484	10	9	1800	599	9.05	67263.68	6	plas, E.R.
DenCslA14	rna-XM_020839336.2	683043	687972	9	8	1413	470	8.49	53993.82	3	plas, golg_plas, vacu
DenCslA15	rna-XM_020839335.2	682851	687972	10	9	1455	484	7.58	55785.62	1	plas, golg_plas, vacu
DenCslA16	rna-XM_020839350.2	714120	720822	10	9	1692	563	9.11	64700.67	6	plas, vacu
DenCslA17	rna-XM_020839343.2	743654	816186	10	9	1644	547	8.56	62599.07	5	plas, vacu, E.R.
DenCslA18	rna-XM_020839341.2	743655	816186	10	9	1671	556	8.56	63590.16	5	plas, cyto_plas
DenCslA19	rna-XM_020839349.2	714120	720825	10	9	1695	564	9.11	64771.75	5	plas, vacu, E.R.
DenCslC1	rna-XM_020837267.2	5881809	5886114	5	4	2055	684	8.81	77994.64	5	plas
DenCslC2	rna-XM_020841438.2	1477345	1480521	5	4	1872	623	8.86	71495.85	6	plas
DenCslC3	rna-XM_020830667.2	354560	365362	5	4	2082	693	9.27	79712.07	6	plas
DenCslC4	rna-XM_020820976.2	5818619	5823745	5	4	2046	681	9.01	77961.13	5	plas
DenCslC5	rna-XM_020848683.2	711393	716795	5	4	2073	690	8.66	78555.59	5	plas
DenCslD1	rna-XM_020844545.2	2993792	2997458	3	2	3120	1039	8.43	115821.55	8	plas
DenCslD2	rna-XM_020823483.2	20378522	20384906	3	2	3423	1140	6.76	127811.67	8	plas
DenCslD3	rna-XM_020849821.2	218232	221190	1	0	2376	791	8.84	88877.51	6	plas, vacu
DenCslD4	rna-XM_020840092.2	1077762	1082979	5	4	2223	740	5.75	82383.96	2	chlo, mito
DenCslD5	rna-XM_028697189.1	492781	495772	2	1	2592	863	8.57	97991.25	7	plas
DenCslD6	rna-XM_020829667.2	1151327	1156560	3	2	3462	1153	6.50	129061.47	8	plas
DenCslD7	rna-XM_020849337.2	739240	743993	5	4	3519	1172	5.68	130380.35	6	plas
DenCslD8	rna-XM_028695875.1	406655	418569	7	6	3543	1180	7.89	131733.04	8	plas
DenCslE1	rna-XM_020837356.2	357017	363143	8	7	2190	729	8.12	83067.58	8	plas
DenCslE2	rna-XM_020837355.2	366228	371432	7	6	1842	613	7.86	70609.91	6	plas
DenCslE3	rna-XM_020837352.2	366228	371432	8	7	2184	727	8.43	83581.36	8	plas
DenCslE4	rna-XM_020837351.2	366232	371432	8	7	2190	729	8.50	83808.67	8	plas
DenCslE5	rna-XM_020837350.2	390655	396565	8	7	2193	730	7.94	83696.62	8	plas
DenCslE6	rna-XM_028698959.1	378096	395593	7	6	1395	464	7.17	54328.52	2	nucl, cyto
DenCslE7	rna-XM_028698958.1	378070	396512	8	7	2226	741	8.20	85761.01	8	plas
DenCslG1	rna-XM_020827350.2	9834	20089	7	6	2139	712	7.18	80943.69	7	plas
DenCslG2	rna-XM_020836250.2	102446	120034	6	5	2142	713	7.16	81316.00	7	plas
DenCslG3	rna-XM_020836251.2	68669	74378	5	4	1248	415	6.24	47275.30	1	chlo
DenCslH1	rna-XM_028695005.1	18470706	18483281	9	8	2310	769	8.80	87093.48	5	plas

Three of the 125 *CesA/Csl* proteins (AsCslA2, PeqCslE4, and PeqCslH3) had extremely short amino acid sequences (less than 300 aa), even after reannotation via Softberry^13^. This may be due to genome sequencing errors or improper assembly. Thus, the three proteins were excluded from the subsequent statistical analysis. The molecular weights of the remaining 122 *CesA/Csls* ranged from 42.84 kDa (PeqCslA9) to 146.86 kDa (AsCslD5), with pI values ranging from 5.63 (DofCesA11) to 9.29 (AsCslA3 and DofCslA4) (Supplementary Table S1). The longest protein was AsCslD5 (1392 aa), whereas the shortest was PeqCslA9/PeqCslA10 (374 aa). The amino acid sequences of the *CesA/Csls* ranged from 415 aa to 1,180 aa in *D. officinale*, 515 aa to 1157 aa in *P. equestris*, and 438 aa to 1329 aa in *A. shenzhenica*. Except for four proteins, most of the CesAs and CslDs in the three orchid species were approximately 800–1,000 aa in length with 6–8 transmembrane (TMs) domains (Supplementary Table S1). The DofCslAs and DofCslCs were generally ∼500 aa and ∼700 aa in length, respectively, with 5 or 6 TM domains. Moreover, subcellular localization prediction showed that 94% of the *CesA/Csls* were localized on the plasma membrane. Nonetheless, two AsCslAs and four DofCslAs were predicted to localize on both the plasma membrane and the Golgi apparatus plasma. Detailed information on the *CesA/Csl* proteins in the three orchid species, including their gene/protein ID, CDS length, subcellular localization, TM domains, molecular weight, and pI value are shown in Table 1 and Supplementary Table S1.

### Phylogenetic Relationships and Gene Expansion of *CesA/Csl* Proteins in Major Plant Species

**FIGURE 2:**
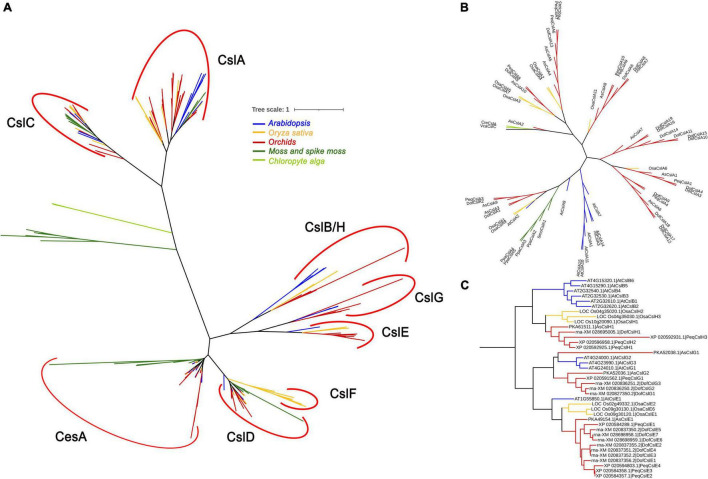
Phylogenetic relationships between *Cellulose synthase* proteins from nine representative species: two chlorophyte algae (light green), a moss and spike moss (dark green), three orchid species (red), rice (yellow), and *Arabidopsis* (blue). The *CesA/Csl* proteins were grouped into nine families: CesA, CslA, CslB, CslC, CslD, CslE, CslF, CslG, and CslH. **(A)** The phylogenetic tree contains all 273 *CesA/Csl* protein sequences; **(B)** phylogenetic tree showing only the CslA cluster; **(C)** phylogenetic tree showing only the CslB/H/E/G cluster. The sequences in **(B,C)** were extracted from **(A)** and realigned.

To investigate the evolution of the *Cellulose synthase* gene superfamily, phylogenetic analysis was performed for nine representative plant and algal species, i.e., two algal species (*Volvox carteri* and *Chlamydomonas reinhardtii*), *Physcomitrella patens*, *Selaginella moellendorffii*, *Oryza sativa*, *Arabidopsis*, and three orchid species (*D. officinale*, *P. equestris*, and *A. shenzhenica)*. A phylogenetic tree was constructed based on 273 amino acid sequences using MEGA 6 software and the maximum likelihood (ML) method with 1,000 bootstraps. The 273 *CesA/Csls* were classified into CesA and eight Csl families: CslA, CslB, CslC, CslD, CslE, CslF, CslG, and CslH (Figure 2A and Supplementary Figure S2). The lower land plants, i.e., the bryophyte *P. patens* and the lycophyte *S. moellendorffii*, are identified with representatives of the CesA, CslA, CslC, and CslD families, which is generally consistent with previous findings (Roberts and Bushoven, 2007; Yin et al., 2014). Each of the two green algae was identified as containing a single copy of the CslA/C-like gene (VcaCslC for *V. carteri* and CreCslA for *C. reinhardtii*), implying that the possible duplication of the common ancestor of CslA/CslC occurred in land plants after they diverged from green algae (Yin et al., 2009; Popper et al., 2011). Among the eight Csl families, CslA and CslC are distantly related to the other Csl families, supporting previous suggestions that the CslA and CslC family members evolved from a separate cyanobacterial endosymbiotic event (Yin et al., 2009). CslB was found only in the dicot species *Arabidopsis*, whereas CslH was specific to the four monocots (Figure 2C). Notably, CslG family members were found in all three Orchidaceae species.

The number of CesAs was rather conserved among angiosperms, with each generally having 8∼13 members (except for *S. moellendorffii*), whereas the members in the other families greatly varied. *P. patens* had the highest number of CesAs (13 members) and CslDs (10 members), whereas *S. moellendorffii* had the lowest (five SmoCesAs and three SmoCslDs). The CslCs were most abundant in *S. moellendorffii* (10 SmoCslCs); however, the family had greatly contracted in other species, especially in *P. equestris* (containing only PeqCslC1). Notably, the CslA family had significantly expanded in *D. officinale* (with 19 DofCslAs) compared with the remaining species (Figure 2B), which may have resulted in functional redundancy or innovation.

### Structural Conservation and Diversity of *CesA/Csls* in Orchidaceae Species

**FIGURE 3:**
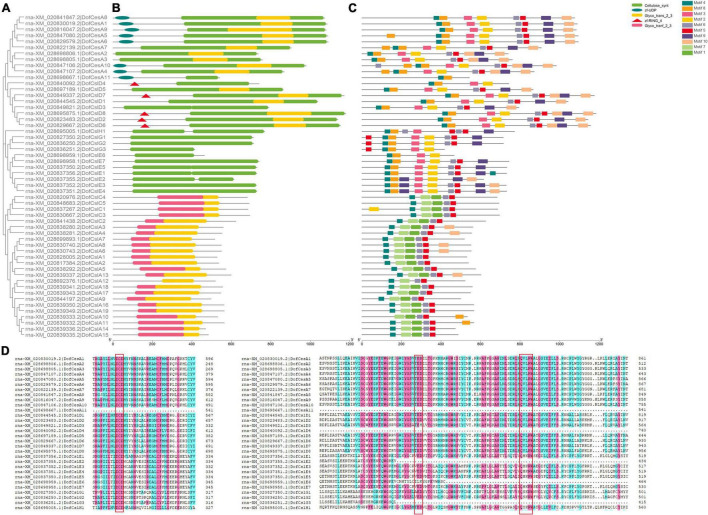
Phylogenetic, conserved domain and motif analyses of *Cellulose synthase* proteins in *D. officinale* (Dof). **(A)** Phylogenetic tree of the DofCesA/DofCsl proteins; **(B)** Schematic presentation of the domains of the DofCesA/DofCsl proteins; **(C)** Schematic presentation of the conserved motifs in the DofCesA/DofCsl proteins; **(D)** Comparison of the deduced amino acid sequences of *Cellulose synthase* in *D. officinale*. The conserved cysteine residues are marked by red frames. The full versions of multiple alignments of the DofCesA/DofCsl protein sequences are shown in Supplementary Figure S5. The scale bar (aa) indicates the amino acid position within the corresponding conserved domain/motif.

The sequences of conserved domains of the 125 *CesA/Csl* proteins in three Orchidaceae species were searched and analyzed. Five conserved domains: *Cellulose synthase* domain (cellulose_synt), zf-UDP, zf-RING_4, and two glycosyltransferase family domains (Glyco_transf_2, Glyco_trans_2_3) were found (Figure 3 and Supplementary Figures S3, S4). We found that the *Cellulose synthase* domain was present in CesA and CslD/E/G/H, whereas all the CslA/CslC proteins were found to contain two glycosyltransferase domains (Table 2), which support the CslA/CslC families evolved from an independent cyanobacterial endosymbiotic event (Yin et al., 2009). The number of CesA and CslD proteins that contain zf-UDP/zf-RING_4 domains varied among the three Orchidaceae species. For example, the zf-UDP domain was found in all nine AsCesA proteins but was absent from three DofCesAs and one PeqCesA. The motif patterns also varied among different species and families; however, we found species-specific motifs and similar conserved motif patterns within the same *CesA/Csl* family (Table 2, Figure 3 and Supplementary Figures S3, S4). These results suggested that *CesA/Csl* proteins among different families in Orchidaceae species might have different functional properties. Furthermore, multiple alignments of the predicted *Cellulose synthase* amino acid sequences showed that 18 of the 125 *CesA/Csl* proteins had no “D,D,D,QxxRW” integrated active site amino acid sequence (Supplementary Figures S5–S7), implying possible functional redundancy.

**TABLE 2:** Structure of the *Cellulose synthase* proteins in *D. officinale*.

Group	Gene_Protein ID	Size (aa)	Domain 1	Position (aa)	Domain 2	Position (aa)
DenCesA1	rna-XM_020830019.2	1086	cellulose_synt	359–1076	zf-UDP	29−106
DenCesA2	rna-XM_028698806.1	737	cellulose_synt	11–726		
DenCesA3	rna-XM_028698805.1	758	cellulose_synt	32–747		
DenCesA4	rna-XM_020847107.2	868	cellulose_synt	142–857	zf-UDP	8−65
DenCesA5	rna-XM_020847080.2	1090	cellulose_synt	357–1082	zf-UDP	29−106
DenCesA6	rna-XM_020829579.2	1091	cellulose_synt	358–1083	zf-UDP	29−106
DenCesA7	rna-XM_020822139.2	904	cellulose_synt	139–897		
DenCesA8	rna-XM_020841847.2	1073	cellulose_synt	345–1067	zf-UDP	12−84
DenCesA9	rna-XM_020816047.2	1081	cellulose_synt	375–1074	zf-UDP	58−134
DenCesA10	rna-XM_020847106.2	980	cellulose_synt	254–969	zf-UDP	8−60
DenCesA11	rna-XM_028698667.1	541	cellulose_synt	368–520	zf-UDP	27−106
DenCslA1	rna-XM_020828005.2	528	Glycos_transf_2	97–253	Glyco_trans_2_3	186−375
DenCslA2	rna-XM_020817384.2	536	Glycos_transf_2	105–263	Glyco_trans_2_3	194−384
DenCslA3	rna-XM_020838280.2	559	Glycos_transf_2	127–287	Glyco_trans_2_3	215−408
DenCslA4	rna-XM_020838281.2	552	Glycos_transf_2	120–281	Glyco_trans_2_3	208−395
DenCslA5	rna-XM_020838292.2	572	Glycos_transf_2	139–284	Glyco_trans_2_3	227−444
DenCslA6	rna-XM_020830743.2	550	Glycos_transf_2	99–259	Glyco_trans_2_3	187−404
DenCslA7	rna-XM_028699893.1	516	Glycos_transf_2	95–254	Glyco_trans_2_3	182−387
DenCslA8	rna-XM_020830740.2	549	Glycos_transf_2	98–258	Glyco_trans_2_3	186−394
DenCslA9	rna-XM_020844197.2	497	Glycos_transf_2	74–223	Glyco_trans_2_3	163−371
DenCslA10	rna-XM_020839333.2	531	Glycos_transf_2	144–257	Glyco_trans_2_3	187−393
DenCslA11	rna-XM_020839332.2	564	Glycos_transf_2	133–290	Glyco_trans_2_3	220−426
DenCslA12	rna-XM_028692376.1	519	Glycos_transf_2	125–257	Glyco_trans_2_3	254−382
DenCslA13	rna-XM_020839337.2	599	Glycos_transf_2	168–323	Glyco_trans_2_3	257−446
DenCslA14	rna-XM_020839336.2	470	Glycos_transf_2	133–291	Glyco_trans_2_3	220−415
DenCslA15	rna-XM_020839335.2	484	Glycos_transf_2	133–291	Glyco_trans_2_3	220−414
DenCslA16	rna-XM_020839350.2	563	Glycos_transf_2	130–289	Glyco_trans_2_3	218−425
DenCslA17	rna-XM_020839343.2	547	Glycos_transf_2	116–276	Glyco_trans_2_3	205−410
DenCslA18	rna-XM_020839341.2	556	Glycos_transf_2	125–285	Glyco_trans_2_3	214−419
DenCslA19	rna-XM_020839349.2	564	Glycos_transf_2	131–290	Glyco_trans_2_3	219−426
DenCslC1	rna-XM_020837267.2	684	Glycos_transf_2	232—387	Glyco_trans_2_3	320−514
DenCslC2	rna-XM_020841438.2	623	Glycos_transf_2	168–327	Glyco_trans_2_3	256−469
DenCslC3	rna-XM_020830667.2	693	Glycos_transf_2	237–411	Glyco_trans_2_3	325−519
DenCslC4	rna-XM_020820976.2	681	Glycos_transf_2	231–386	Glyco_trans_2_3	319−514
DenCslC5	rna-XM_020848683.2	690	Glycos_transf_2	237–411	Glyco_trans_2_3	325−520
DenCslD1	rna-XM_020844545.2	1039	cellulose_synt	280–1029		
DenCslD2	rna-XM_020823483.2	1140	cellulose_synt	366–1131	zf-RING_4	122−171
DenCslD3	rna-XM_020849821.2	791	cellulose_synt	54–395, 408–781		
DenCslD4	rna-XM_020840092.2	740	cellulose_synt	320–675	zf-RING_4	86−134
DenCslD5	rna-XM_028697189.1	863	cellulose_synt	94–856		
DenCslD6	rna-XM_020829667.2	1153	cellulose_synt	386–1143	zf-RING_4	138−186
DenCslD7	rna-XM_020849337.2	1172	cellulose_synt	405–1155	zf-RING_4	143−191
DenCslD8	rna-XM_028695875.1	1180	cellulose_synt	389–1170	zf-RING_4	135−184
DenCslE1	rna-XM_020837356.2	729	cellulose_synt	98–388, 417–706		
DenCslE2	rna-XM_020837355.2	613	cellulose_synt	98–396, 435–601		
DenCslE3	rna-XM_020837352.2	727	cellulose_synt	98–394, 408–715		
DenCslE4	rna-XM_020837351.2	729	cellulose_synt	98–396, 409–717		
DenCslE5	rna-XM_020837350.2	730	cellulose_synt	98–393, 406–671		
DenCslE6	rna-XM_028698959.1	464	cellulose_synt	109–404		
DenCslE7	rna-XM_028698958.1	741	cellulose_synt	109–404, 416–682		
DenCslG1	rna-XM_020827350.2	712	cellulose_synt	87–383, 394–703		
DenCslG2	rna-XM_020836250.2	713	cellulose_synt	87–386, 394–706		
DenCslG3	rna-XM_020836251.2	415	cellulose_synt	86–385		
DenCslH1	rna-XM_028695005.1	769	cellulose_synt	98–360, 414–763		

To investigate the sequence diversity of the *CesA/Csl* proteins in the three orchid species, putative motifs were identified using MEME Suite 5.0.2; a total of 10 conserved motifs were found (Figure 3C and Supplementary Figures S3, S4). Although the motifs varied among the different species and families, the *CesA/Csl* proteins in each family in the same species shared several unique motifs. For example, in *D. officinale*, motif4, motif8, and motif2 were all observed in the DofCesA, DofCslD, DofCslG, and DofCslE families but not in the DofCesA11 family (Figure 3C and Supplementary Figure S5A). The DofCslA and DofCslC family proteins shared five common motifs: motif4, motif7, motif1, motif6, and motif5; however, DofCslA10 lacked motif 4. Among these motifs, motif7 and motif1 were only observed in DofCslA and DofCslC families. In *P. equestris*, the conserved motifs in PeqCesA, PeqCslD, PeqCslE, PeqCslH, and PeqCslG were similar; except for four proteins, all contained motif1–motif7 and motif10 (Supplementary Figure S3). Moreover, motif9 was specific to members of the PeqCslA and PeqCslC families. In *A. shenzhenica*, motif1, motif5, and motif2 were uniformly observed in all *CesA/Csl* proteins, with the exceptions of AsCesA9 and AsCslA2. Members of AsCesA, AsCslD, AsCslE, AsCslH, and AsCslG also shared similar motifs (Supplementary Figure S4), and motif 10 was specifically present in the AsCslA and AsCslC proteins. Taken together, these results revealed species-specific motifs and similar motifs shared among certain families. Thus, *CesA/Csl* proteins among different families and species might have different functional properties.

### Gene Structure, Distribution, and Duplication Analyses

**FIGURE 4:**
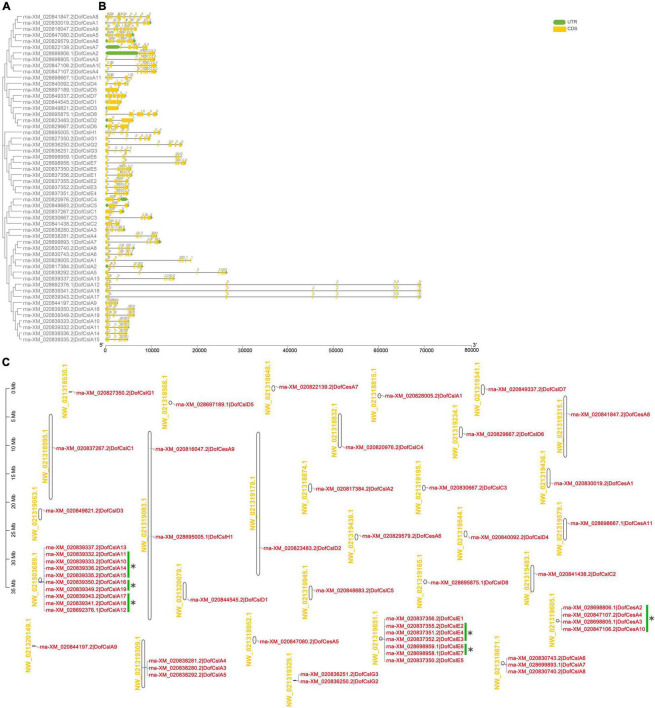
Phylogenetic, gene structure, and distribution analyses of *Cellulose synthase*superfamily genes in *D. officinale* (*Dof*). **(A)** Phylogenetic relationships of the *DofCesA/DofCsl* genes; **(B)** Sequence analysis of exon–intron structures of the *Cellulose synthase* genes. UTRs and exons are indicated using green and yellow rectangles, respectively. The solid lines indicate introns. The numbers above the solid lines represent the intron phase. The scale bar (bp) indicates the length of the corresponding genes; **(C)** distribution of the*Cellulose synthase*genes in the *D. officinale* genome. The scaffold names are shown on the left, and the gene names are on the right. The scale bar (Mb) indicates the length of the corresponding scaffolds. A total of 14 tandemly duplicated genes (with two or more homologous genes ≤ 100 kb apart) were found. Green scale bars and asterisks indicated alternative splicing genes.

To gain more information on the evolutionary patterns of *Cellulose synthase* genes, the gene structures of the *CesA/Csl*s in *D. officinale*, *P. equestris*, and *A. shenzhenica* were analyzed (Supplementary Table S1). The intron–exon structure of the *CesA/Csls* was highly diverse among the different families within the same species (Figures 4A,B). Nonetheless, the same family members have similar exon/intron numbers among the three Orchidaceae species. For example, except for four genes, *AsCesAs* and *PeqCesAs* usually have 13 or 14 exons. The exon number in *DenCesAs* varied more significantly compared to the other two orchids and ranged from 8 to 16 exons. *CslAs* generally contain 5–10 exons, and most have 9 or 10 exons. The exon–intron structures and intron phases of the genes were relatively conserved within the same family (Figure 4B and Supplementary Figures S3, S4). In *D. officinale*, all of the *DofCslCs* had 5 exons and 4 introns. The gene structure varied the most within the *CslD* genes, from 0–7 or 8 introns/exons. These results suggested that possible functional diversity occurred within the same family and evolutionary conservation in Orchidaceae species.

Based on the physical positions of the *Cellulose synthase* genes in the *D. officinale* genome (Zhang et al., 2016), the distribution of 54 *DofCesA/DofCsl* genes was mapped to 30 scaffolds (Figure 4C and Table 1). Among them, 23 scaffolds had only one *Cellulose synthase* gene and two scaffolds each had two genes. Apart from the alternative splicing genes (indicated with green scale bars and asterisks), 14 tandemly duplicated genes (with two or more homologous genes ≤ 100 kb apart) were detected on six scaffolds (Figure 4C). A maximum number of 10 genes were detected on scaffold NW_021503689.1, all of which were *DofCslA* members (*DofCslA10*-*19*); after merging alternative splicing genes, there were four tandemly duplicated genes. DofCslA3, -4, -5 and DofCslA6, -7, -8 were also tandem duplicated genes located on scaffold NW_021319309.1 and NW_021319871.1, respectively. In addition, the *DofCslEs* also contain tandemly duplicated members that clustered on scaffold NW_02131965.1 (Figure 4C). Moreover, we found that the structures of alternative splicing genes, such as the number of exons and the length of introns, varied in different degrees among the splicing variants of the same gene (Supplementary Figure S8 and Supplementary Table S2). These results revealed that the expansion of the *DofCesA/DofCsl* genes mainly originated through gene duplication.

### Diverse Expression Patterns of *DofCesA/DofCsl* Genes in *Dendrobium officinale*

**FIGURE 5:**
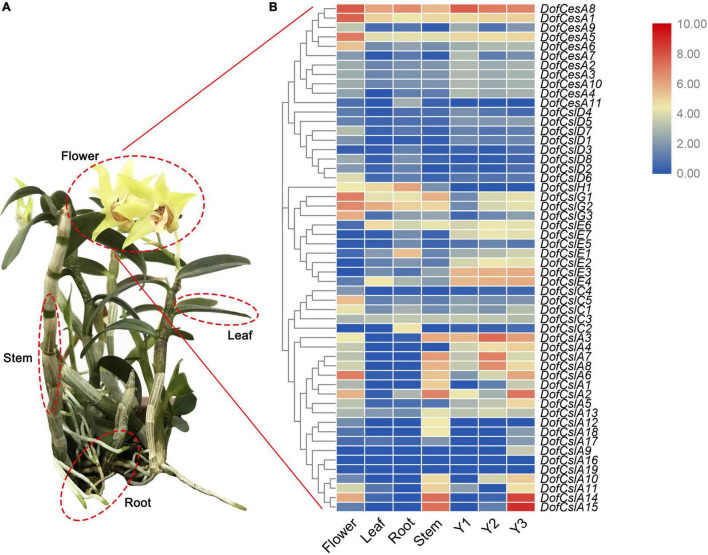
Hierarchical clustering of the expression profiles of *Cellulose synthase* superfamily genes in different organs (flowers, roots, stems, and leaves) of *D. officinale* and at different growth stages (Y1, Y2, and Y3). **(A)** Image of a 3-year-old *D. officinale* plant; **(B)** FPKM values visualized as the heatmap.

To analyze the roles of the *CesA/Csl* genes in *D. officinale*, their expression patterns were investigated in different organs in *D. officinale* at different developmental stages. Transcriptome sequencing was performed on four organs of *D. officinale* (i.e., the leave, stem, flower, and root) and the stems of three different stages (Y1, Y2, and Y3). The expression levels of the *DofCesA/DofCsl* genes are presented in heatmaps in Figure 5 and Supplementary Table S3). The results revealed diverse expression patterns of the *DofCesA/DofCsl* genes, suggesting functional divergence had occurred after gene expansion.

In the *DofCesA* family, three genes (*DofCesA1*, *DofCesA5*, and *DofCesA8*) were consistently expressed across different organs and developmental stages and were all significantly expressed in the flowers. Among them, *DofCesA8* also had high expression levels in the roots and low expression in the leaves and stems; both *DofCesA1* and *DofCesA5* were expressed at low levels in the leaves, roots, and stems. Notably, *DofCesA8* was also highly expressed in the Y1, Y2, and Y3 stems of *D. officinale*. The *DofCslD* and *DofCslC* family genes exhibited similar expression patterns, with low expression across the four organs and three developmental stages. The *DofCslEs* showed organ-specific expression patterns; for example, *DofCslE1* and *DofCslE4* exhibited moderate expression in the roots and leaves, respectively, whereas *DofCslE6* was expressed in the leaves, roots, and stems and across all three growth stages. *DofCslH1* was expressed in three organs (flowers, leaves, and roots), with the highest expression level in the roots. Moreover, the *DofCslG* family genes showed similar expression patterns; all exhibited higher expression levels in the flowers than in the roots, leaves, and stems. *DofCslG1* and *DofCslG2* also exhibited moderate expression in Y2 and Y3 stems.

*CslAs* are known for their participation in the synthesis of polysaccharides, especially mannans and glucomannans. Interestingly, the *DofCslAs* exhibited apparent organ- and development-specific expression in *D. officinale*. Most of these genes were predominantly expressed in the stems and/or flowers, with very low levels in the roots and leaves. For example, *DofCslA2*, *DofCslA14*, and *DofCslA15* had significantly higher expression levels in the stems than in the other organs (especially *DofCslA15*); *DofCslA2* and *DofCslA14* were also expressed in the flowers. However, several genes had almost no expression in any of the four organs. In addition, *DofCslA6* was specifically expressed in the flowers. Among the three growth stages, some genes showed similar expression patterns, such as *DofCslA7* and *DofCslA8*, both of which were expressed at higher levels in the Y2 stems. Notably, *DofCslA14* and *DofCslA15* had significantly high expression in Y3 stems. However, five of the 19 *DofCslAs* showed low levels of or no expression in the Y1, Y2, and Y3 stems. *DofCslA2* was expressed at moderate levels in the Y3 stems and at low levels in the Y1 stems, whereas *DofCslA3* showed a consistent expression pattern across all three stages, with the highest expression levels occurring in Y2 stems. These results revealed the possible functional specialization of *DofCslAs* in the polysaccharide synthesis in *D. officinale*.

### Water-Soluble Polysaccharide Contents in *Dendrobium officinale*

**FIGURE 6:**
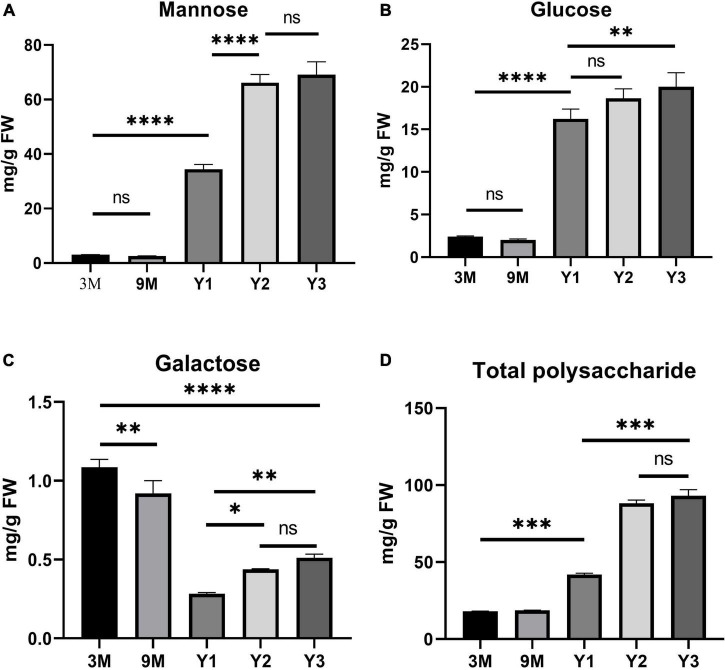
Histogram of the contents of water-soluble polysaccharides and monosaccharide components (mg/g) in the stems of *D. officinale* in different growth stages, including 3M, 9M, Y1, Y2, and Y3. **(A)** Mannose content; **(B)** glucose content; **(C)** galactose content; **(D)** total polysaccharide content. *p value < 0.05, **p value < 0.01, ***p value < 0.001, ****p value < 0.0001.

A previous study showed that polysaccharide accumulation in *D. officinale* occurred predominantly in the stems (He et al., 2015). To further investigate the polysaccharide synthesis and functions of *Cellulose synthase* superfamily genes in Orchidaceae species, we measured the contents of total water-soluble polysaccharides, and their component monosaccharides (glucose, mannose, and galactose) in the stems of *D. officinale* at five different growth stages, i.e., 3 months (3M), 9 months (9M), 1 year (Y1), 2 years (Y2), and 3 years (Y3) (Figure 6). The results showed that the total polysaccharide content varied significantly among the different stages (Figure 6D and Table 3); it was lowest in the 3M stems (∼18.06 mg/g) and highest in the Y3 stems (∼93.02 mg/g). The total polysaccharide contents varied slightly between the 3M and 9M stems and between Y2 and Y3 stems, but increased significantly from 3M/9M to Y2/Y3.

**TABLE 3:** Contents of water-soluble polysaccharides and constituting monosaccharide (mg/g) in the stems of *D. officinale* at different growth stages, including 3M, 9M, Y1, Y2, and Y3.

Sample	Mannose (mg/mL)	Mean	Galactose (mg/mL)	Mean	Glucose (mg/g FW)	Mean	Total (mg/g FW)	Mean
3M	3.0796	3.0917	1.0831	1.0854	2.3171	2.4017	18.1847	18.0641
	3.0656		1.1368		2.5021		17.9997	
	3.1299		1.0362		2.3860		18.0077	
9M	2.5761	2.5889	0.8282	0.9193	1.9033	2.0086	18.6568	18.6748
	2.6111		0.9844		2.1444		18.5024	
	2.5794		0.9453		1.9782		18.8652	
Y1	35.5856	34.4407	0.2770	0.2827	16.2450	16.2441	42.4609	41.8861
	35.2837		0.2910		17.3928		42.3283	
	32.4527		0.2800		15.0945		40.8691	
Y2	67.5208	66.1652	0.4390	0.4383	17.7653	18.6345	88.3224	88.2296
	68.2550		0.4410		19.9196		90.2813	
	62.7199		0.4350		18.2186		86.0851	
Y3	73.0744	69.1258	0.5370	0.5120	21.8808	20.0141	96.1469	93.0240
	70.4218		0.4960		19.3982		94.5241	
	63.8812		0.5030		18.7632		88.4010	

To determine the composition and contents of monosaccharides in the stems, the neutral monosaccharide composition was analyzed at the five growth stages. The results revealed that mannose was the most abundant neutral monosaccharide in the stems of *D. officinale* across all five growth stages, followed by glucose and galactose (Figure 6). The contents of glucose and mannose ranged from 2.40 to 20.01 mg/g and from 3.09 to 69.13 mg/g, respectively, with the highest detected in the Y3 stems and the lowest in the 3M stems. Mannose and glucose in the stems increased remarkably during *D. officinale* development, corresponding to the increases in total polysaccharides. Interestingly, the galactose content was highest in the 3M stems; however, this content decreased as *D. officinale* developed, lowest in the Y1 stems, and then increased slightly from Y2 to Y3.

### qPCR Analyses of *DofCslA* Genes in the Stems of *Dendrobium officinale* at Different Developmental Stages

**FIGURE 7:**
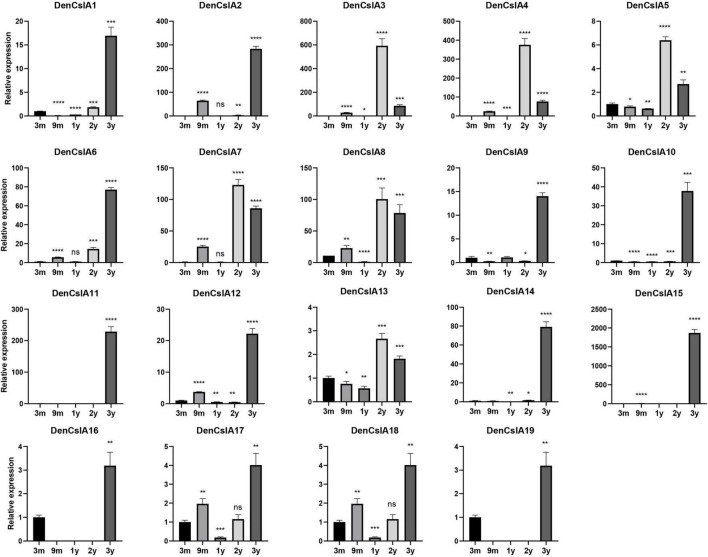
Expression levels of *DofCslA* genes in the stems of *D. officinale* in different growth stages (including 3M, 9M, Y1, Y2, and Y3), as determined by qRT–PCR analysis. The results are shown as the means ± SDs of three independent experiments. The presented expression levels are relative to the expression of the reference gene. *p value < 0.05, **p value < 0.01, ***p value < 0.001, ****p value < 0.0001.

To better understand the roles of *CslAs* in polysaccharide synthesis in *D. officinale*, qPCR analyses of the 19 *DofCslAs* were performed on *D. officinale* stems at five different growth stages: 3M, 9M, Y1, Y2, and Y3. The results for Y1, Y2, and Y3 were generally consistent with the transcriptome sequencing data. In general, most of the *DofCslAs* exhibited high expression levels in Y2 and/or Y3 stems (Figure 7 and Supplementary Table S4), which was in agreement with the accumulation of water-soluble polysaccharides and monosaccharides. Among them, five genes, i.e., *DofCslA2*, *DofCslA6*, *DofCslA11*, *DofCslA14*, and *DofCslA15*, exhibited significantly high expression levels in the Y3 stems (especially *DofCslA15*). Four genes, i.e., *DofCslA3*, *DofCslA4*, *DofCslA7*, and *DofCslA8*, were highly expressed in the Y2 stems compared with the Y3 stems. Notably, several genes, such as *DofCslA2*, *DofCslA3*, and *DofCslA7*, were also expressed in the 9M stems; *DofCslA8* also exhibited moderate expression in the 3M stems. In addition, six *DofCslAs*, such as *DofCslA16-19*, showed low expression levels. The primer sequences for qPCR analysis are provided in Supplementary Table S5. Taken together, these results suggest that *DofCslA* genes may exhibit functional redundancy and may be specialized for activity at different growth stages.

## Discussion

Owing to its important roles in the biosynthesis of plant cell wall polysaccharides, primarily cellulose and hemicellulose, the *Cellulose synthase* superfamily has been the subject of intense studies. Much effort has been devoted to the identification of the *CesA/Csl* family in plants, including mosses, lycophytes, monocots, and dicots, as well as in green algae (Persson et al., 2007; Yin et al., 2014; Takata and Taniguchi, 2015; Li et al., 2017, 2020; Song et al., 2018; Cui et al., 2020). However, knowledge of the *Cellulose synthase* superfamily is still lacking in the family Orchidaceae. In the present study, we performed genome-wide characterizations of *CesA/Csl* family members in three fully sequenced Orchidaceae species (Cai et al., 2014; Zhang et al., 2016, 2017), which revealed the evolution, structural divergence, and expression profiles of *CesA/Csls*.

### Species- and Family-Specific Expansion of *CesA/Csl* Proteins

The *Cellulose synthase* superfamily belongs to the glycosyltransferase-2 superfamily, which is widely distributed throughout the plant kingdom (Kim et al., 2007; Cantarel et al., 2009). In the present study, we identified a total of 125 *Cellulose synthase* superfamily members from the three sequenced orchids using two Pfam domains (PF03552/PF00535; Table 1 and Supplementary Table S1). Among them, 54 DofCesA/DofCsls, 37 PeqCesA/PeqCsls, and 34 AsCesA/AsCsls were identified in the genomes of *D. officinale*, *P. equestris*, and *A. shenzhenica*, respectively. The results revealed species-specific expansion of the *Cellulose synthase* superfamily in *D. officinale*.

In land plants, the *Cellulose synthase* superfamily can be further classified into a CesA family and nine Csl families: CslA-CslH and CslJ (Fincher, 2009; Yin et al., 2014; Schwerdt et al., 2015). Previous studies indicate that PF03552 encompasses the CesA family and seven Csl families (CslB/D/E/F/G/H/J), whereas PF03552 is absent from the CslA/CslC families (Yin et al., 2009). In the present study, the CesA superfamily genes in Orchidaceae were grouped into one CesA family and six Csl families: CslA, CslC, CslD, CslE, CslG, and CslH (Figure 1 and Supplementary Figure S1). The number of CesAs in the three orchid species was largely the same, while the number of Csls greatly varied, from 25 AsCsls to 43 DofCsls. Interestingly, the Csl proteins exhibited family-specific expansion among the three orchid species; for example, there were 19 DofCslAs, almost twice that in the other two orchids. The results of similar expansion were also observed in Csl families such as PeqCslDs and PeqCslHs. The expansion of *CesA/Csl* families likely resulted in functional redundancy or innovation. To further investigate the evolution of *CesA/Csls*, a phylogenetic tree based on 273 amino acid sequences from nine representative plant species was constructed (Figure 2). *CesA/Csls* were classified into one CesA family and eight Csl families: CslA, CslB, CslC, CslD, CslE, CslF, CslG, and CslH. These results are consistent with those of previous findings in which CslA, CslC, and CslD were found to be conserved in all land plants (Farrokhi et al., 2006; Yin et al., 2014). CslF and CslH may be restricted to monocots, and CslFs were absent from the three Orchidaceae species. CslG members were previously thought to be confined to eudicots (Fincher, 2009). However, CslG family members were found in all three Orchidaceae species, supporting the proposal that CslG should no longer be considered a dicot-specific family (Yin et al., 2014). In addition, our results strongly supported previous findings that the numbers of *CesA/Csls* vary greatly among different plant species (Paterson et al., 2009; Takata and Taniguchi, 2015; Song et al., 2018; Zou et al., 2018; Li et al., 2020). The number of CesAs is largely conserved among angiosperms, most of which have 8–13 members (except for *S. moellendorffii*), whereas the members in other families greatly varied among different species, indicating that extensive expansion and diversification have occurred.

We identified three proteins in *P. equestris* and *A. shenzhenica* that had extremely short amino acid sequences (less than 300 aa). The molecular weights of the remaining 122 *CesA/Csls* in the three orchid species ranged from 42.84 to 146.86 kDa. The longest protein was AsCslD5-1392 aa, which is shorter than the 2038 aa sequence in tomato (Song et al., 2018). Previous studies have indicated that cellulose is synthesized by plasma membrane-localized *Cellulose synthase* complexes with access to the cytosolic GDP-glucose pool (Taylor, 2008; Endler and Persson, 2011; McFarlane et al., 2014; Schneider et al., 2016). In the present study, subcellular localization predictions showed that most of the *CesA/Csls* were located on the plasma membrane.

### Sequence Conservation and Diversification of *CesA/Csl* Proteins in Orchids

The GT2 superfamily is characterized by conserved cytosolic substrate binding and catalytic residues. These residues are positioned in a loop between TM domains 2 and 3 and contain a D,D,D,QXXRW motif (Pear et al., 1996). In addition, plant CesA proteins also contain an extended N-terminal zinc-finger domain and two insertions in the catalytic loop, which are presumably involved in specific functions in higher plants, such as multimerization (Pear et al., 1996; Sethaphong et al., 2013). In the present study, five conserved domains were identified from the 125 orchid *CesA/Csls* (Figure 3 and Supplementary Figures S3, S4). We found that the *Cellulose synthase* domain was present in CesA and CslD/E/G/H, whereas all the CslA/CslC proteins were found to contain two glycosyltransferase domains, which support the CslA/CslC families evolved from an independent cyanobacterial endosymbiotic event (Yin et al., 2009). The number of CesA and CslD proteins that contain zf-UDP/zf-RING_4 domains varied among the three Orchidaceae species. For example, the zf-UDP domain was found in all nine AsCesA proteins but was absent from three DofCesAs and one PeqCesA. The motif patterns also varied among different species and families; however, we found species-specific motifs and similar conserved motif patterns within the same *CesA/Csl* family (Figure 3 and Supplementary Figures S3, S4). These results suggested that *CesA/Csl* proteins among different families in Orchidaceae species might have different functional properties. Furthermore, multiple alignments of the predicted *Cellulose synthase* amino acid sequences showed that 18 of the 125 *CesA/Csl* proteins had no “D,D,D,QxxRW” integrated active site amino acid sequence (Supplementary Figures S5–S7), implying possible functional redundancy.

Gene structure and duplication analysis can facilitate the understanding of gene evolution, structural divergence, and functional conservation within a gene family (Worberg et al., 2008; Xu et al., 2012; Nawaz et al., 2017). We found highly diverse structures among the different families but similar exon/intron numbers in the same family among the three orchid species (Figure 4B). The results revealed that there was functional diversity within the same family and that evolutionary conservation occurred among different orchid species. Moreover, a total of 14 tandemly duplicated genes were detected (Figure 4C and Table 1), indicating that the expansion of *DofCesA/DofCsl* genes might result from tandem duplication. These results provide insight into the evolutionary divergence and origins of *CesA/Csl* in Orchidaceae species.

### Functional Divergence of *DofCesA/DofCsl* Genes in Different Organs and Growth Stages of *Dendrobium officinale*

*Cellulose synthase* superfamily genes are involved in the synthesis of the subunits of cellulose and hemicellulose (Burton et al., 2004; Scheller and Ulvskov, 2010; Takata and Taniguchi, 2015). The expansion and diversification of the plant *CesA* superfamily are linked tightly with major events in the evolution of plant and algal lineages, including multicellularity, terrestrialization, and vascularization (Popper et al., 2011). To understand the functions of the *CesA/Csl* genes in orchids, we investigated gene expression profiles in different organs of *D. officinale* and at different developmental stages (Figure 5). The results revealed diverse expression patterns of the *DofCesA/DofCsl* genes, suggesting functional divergence had occurred after gene expansion.

Three *DofCesAs* (*DofCesA1*, *DofCesA5*, and *DofCesA8*) were significantly expressed in the flowers of *D. officinale*. *DofCesA8* also exhibited high expression in the roots and moderate expression in the leaves and stems. Except for *DofCesA6*, which exhibited low expression in the flowers, the expression levels of the remaining *DofCesAs* were minimal in all the tested organs. In addition, *DofCesA8* was also highly expressed in Y1 stems and moderately expressed in the Y2 and Y3 stems. In the *Arabidopsis* genome, ten *AtCesA* genes were identified and found to be responsible for primary (*CesA1-3*, *-5*, *-6*, and *-9*) and secondary (*AtCesA4*, *-7*, and *-8*) cell wall synthesis (Taylor et al., 2003; Burton et al., 2004; Takata and Taniguchi, 2015). Mutations in secondary cell wall *AtCesAs* result in collapsed vasculature or irregular xylem phenotypes (Turner and Somerville, 1997; Taylor et al., 2003). Moreover, *AtCesA1* and *AtCesA3* also play roles in flowering; mutations in *atcesa1* and *atcesa3* result in gamete lethal phenotypes, whereas single-knockout *AtCesA2*, *-5*, *-6*, and *-9* mutants exhibit more moderate phenotypes (Scheible et al., 2001; Caño-Delgado et al., 2003). The *AtCesA1* was clustered in the same subgroup with *DofCesA1*, and *AtCesA3* was clustered with *DofCesA8*. Therefore, the results, combined with the expression patterns, suggested that the three *DofCesAs* likely play roles in flower organ development, and that *DofCesA8* may play additional roles in cellulose deposition in the stems of *D. officinale*.

In plants, the synthesis of hemicellulose occurs in the Golgi membrane by proteins encoded by *Csl* genes (Kaur et al., 2017). In land plants, *CslAs* and *CslCs* encode proteins that are involved in the synthesis of mannan and the β-1,4-linked glucan backbone of xyloglucan, respectively (Liepman et al., 2005, 2010; Cocuron et al., 2007). Moreover, *CslD* members have been reported to have mannan and *Cellulose synthase* activities, especially in tip-growing root hairs and pollen tubes of plant species such as rice and Arabidopsis. In rice, the *OsCSLD1* gene is required for root hair morphogenesis (Kim et al., 2007). *AtCslD3* also plays a role in *Cellulose synthesis*, as *atcsld3* mutants exhibit defects in polarized growth of root hairs and abnormal distribution of cellulose and xyloglucan (Pauly et al., 2001; Park et al., 2011). *CslC*s are involved in the biosynthesis of the β-1,4-linked glucan backbone of xyloglucan (Cocuron et al., 2007). However, the expression of the *DofCslD* and *DofCslC* family genes was either low or absent from the four organs and across the three developmental stages, indicating minimal effects on polysaccharide synthesis in *D. officinale*. Interestingly, most of the *DofCslAs* were predominantly expressed in the stems and/or flowers. *DofCslA2*, *-14*, and *-15* had significantly high expression levels in the stem; *DofCslA6* was specifically expressed in the flowers. Moreover, *DofCslAs* were differentially expressed among the three growth stages. For example, *DofCslA14* and *DofCslA15* exhibited significantly high expression in Y3 stems, while *DofCslA7* and *DofCslA8* were expressed at higher levels in Y2 stems. Five of the 19 *DofCslAs* exhibited low expression or none in the stems of plants at all three growth stages. These results revealed functional divergence and possible redundancy of the *DofCslAs* involved in polysaccharide synthesis in *D. officinale*. The functional role of the CslB/E/G families remains unclear, but *CslF/H* genes have been suggested to be involved in the synthesis of mixed-linkage glucans (MLGs) (Prasad et al., 2005; Burton et al., 2006). We found that the *DofCslGs* all presented higher expression levels in the flowers than in the rest of the organs. *DofCslG1* and *DofCslG2* also had moderate expression in Y2 and Y3 stems. Our phylogenetic study showed that *DofCslE* had significantly expanded in *D. officinale* compared with other species (Figure 2). However, the expression of most *DofCslEs* was very low in different organs and growth stages. Thus, further investigations are needed to elucidate the exact roles of *DofCslE/G/H* family genes.

### Polysaccharide Accumulation and Roles of *DofCslAs* in the Stems of *Dendrobium officinale*

Containing approximately 1,450 species, *Dendrobium* is one of the largest genera of the Orchidaceae family (Zhang et al., 2016). *Dendrobium* species are characterized by their diverse growth habits and bioactive constituents with immunomodulatory hepatoprotective activities, such as dendrobine and polysaccharides (Ng et al., 2012). *D. officinale* stems contain abundant polysaccharides, primarily those consisting of GM and GGM (Xing et al., 2014). The high amount of polysaccharides in *D. officinale* stems supposedly helps maintain osmotic pressure and improve drought tolerance, as this species is epiphytic in natural habitats (Zotz and Tyree, 1996; Wu et al., 2009). *CslAs* encode proteins with both mannan and glucomannan synthase activity (Liepman et al., 2005, 2010). In *Arabidopsis*, the stems of *csla* knockout mutants have no glucomannan, indicating an exclusive role in mannan biosynthesis of the *CslA* family genes (Goubet et al., 2009). To determine the roles of the 19 *DofCslAs* in polysaccharide accumulation in *D. officinale* stems across different growth stages, we performed qRT–PCR and measured the content of total polysaccharides and their monosaccharide components in the stems of 3M, 9M, Y1, Y2, and Y3 plants. The total polysaccharide content was lowest in the 3M stems and highest in the Y3 stems (Figure 6 and Table 3). The content varied slightly between 3M and 9M stems but increased significantly in the mature stems. Monosaccharide composition analysis showed that mannose was the most abundant component across all development stages, supporting previous findings in *Dendrobium* species (Meng et al., 2013). The amounts of glucose and mannose in the stems also experienced remarkable increases from 3M to Y3. In agreement with the changes in total polysaccharide and monosaccharide component contents, most of the *DofCslAs* exhibited high expression levels in the Y2 and/or Y3 stems (Figure 7). Among them, five genes exhibited significantly high expression levels in Y3 stems (especially *DofCslA15*); four genes were highly expressed in Y2 stems. Thus, these genes likely play roles in the biosynthesis of mannan and glucomannan in the stems of *D. officinale*. In addition, six genes showed low expression levels, suggesting that possible redundancy occurred after gene expansion. These results imply that the *DofCslAs* may experience functional specialization with respect to polysaccharide accumulation in different growth stages.

## Data Availability Statement

The raw data of RNA-seq experiment is deposited in Sequence Read Archive (NCBI): PRJNA680456 (https://www.ncbi.nlm.nih.gov/bioproject/PRJNA680456) and PRJNA762115 (https://www.ncbi.nlm.nih.gov/bioproject/PRJNA762115). All data and material used in this study are available from the corresponding author upon reasonable request.

## Author Contributions

YW and CS conceived and designed the experiments, performed the experiments, analyzed the data, prepared the figures and/or tables, authored or reviewed the drafts of the study, and approved the final draft. KZ and YC analyzed the data, authored or reviewed the drafts of the study, and approved the final draft. XC, QW, and HW analyzed the data, contributed reagents, materials, and analysis tools, authored or reviewed the drafts of the study, and approved the final draft. CS conceived the experiments, authored or reviewed the drafts of the study, and approved the final draft. All authors contributed to the article and approved the submitted version.

## Conflict of Interest

The authors declare that the research was conducted in the absence of any commercial or financial relationships that could be construed as a potential conflict of interest.

## Publisher’s Note

All claims expressed in this article are solely those of the authors and do not necessarily represent those of their affiliated organizations, or those of the publisher, the editors and the reviewers. Any product that may be evaluated in this article, or claim that may be made by its manufacturer, is not guaranteed or endorsed by the publisher.

## Footnotes

1
https://www.ncbi.nlm.nih.gov

2
http://tigrfams.jcvi.org/cgi-bin/index.cgi

3
https://web.expasy.org/compute_pi/

4
http://www.cbs.dtu.dk/services/TMHMM/

5
https://wolfpsort.hgc.jp/

6
https://phytozome.jgi.doe.gov/

7
https://itol.embl.de/index.shtml

8
http://gsds.cbi.pku.edu.cn/

9
https://www.ncbi.nlm.nih.gov/Structure/bwrpsb/bwrpsb.cgi

10
http://meme.nbcr.net/meme/

11
http://cole-trapnell-lab.github.io/cufflinks

12
http://hemi.biocuckoo.org/

13
http://www.softberry.com/

## Supplementary Material

Supplementary Figure S1 Phylogenetic tree of the *Cellulose synthase* superfamily members in *D. officinale* (*Dof*), *A. shenzhenica* (*Apo*), and *P. equestris* (*Peq*). The phylogenetic tree was constructed using MEGA 6.0 with the neighbor-joining (NJ) method and 1000 bootstrap replicates. The *CesA/Csl* proteins were grouped into one CesA family and six Csl families: CslA, CslC, CslD, CslE, CslG, and CslH. The families are marked by different arc lines and branch colors. The *D. officinale*, *A. shenzhenica*, and *P. equestris* are distinguished by red, green and blue rhombuses, respectively.

Supplementary Figure S2 Phylogenetic tree of *Cellulose synthase* superfamily members from nine representative plant species. The phylogenetic tree was constructed using MEGA 6.0 with the neighbor-joining (NJ) method and 1000 bootstrap replicates. The *CesA/Csl* proteins were grouped into nine families: CesA, CslA, CslB, CslC, CslD, CslE, CslF, CslG, and CslH. The species included (with the gene code prefixes shown in parentheses) are as follows: *Chlamydomonas reinhardtii* (*Cre*), *Volvox carteri* (*Vca*), *Physcomitrella patens* (*Ppa*), *Selaginella moellendorffii* (*Smo*), *A. shenzhenica* (*Apo*), *D. officinale* (*Den*), *P. equestris* (*Peq*), *Oryza sativa* (*Osa*), and *Arabidopsis thaliana* (*AT*). The families are marked by different arc lines and branch colors.

Supplementary Figure S3 Phylogenetic, conserved domain and motif and gene structure analyses of *Cellulose synthase* proteins in *P. equestris* (*Peq*). **(A)** Phylogenetic relationships and conserved domains of the PeqCesA/PeqCsl proteins. The scale bar (aa) indicates the amino acid position in the corresponding conserved domain. **(B)** Phylogenetic relationships, conserved motifs and gene structure of *PeqCesA/PeqCsl* genes. UTRs and exons are indicated using green and yellow rectangles, respectively. The solid lines indicate introns. The numbers above the solid lines represent the intron phase. Scale bars (bp/aa) indicate the length/amino acid position of corresponding genes/proteins; **(C)** Sequence logos of the 10 motifs.

Supplementary Figure S4 Phylogenetic, conserved domain and motif and gene structure analyses of *Cellulose synthase* proteins in *A. shenzhenica* (*Apo*). **(A)** Phylogenetic relationships and conserved domains of the AsCesA/AsCsl proteins. The scale bar (aa) indicates the amino acid position in the corresponding conserved domains. **(B)** Phylogenetic relationships, conserved motifs and gene structure of the *AsCesA/AsCsl* genes. Exons are indicated in yellow rectangles. The solid lines indicate introns. The numbers above the solid lines represent the intron phase. The scale bars (bp/aa) indicate the length/amino acid position of corresponding genes/proteins; **(C)** sequence logos of the 10 motifs.

Supplementary Figure S5 Multiple alignment and motif logos of *D. officinale*. **(A)** Multiple comparisons of the deduced amino acid sequences of *Cellulose synthase* in *D. officinale*. Conserved cysteine residues are marked by red frames. **(B)** Sequence logos of the 10 motifs.

Supplementary Figure S6 Multiple alignment of the deduced amino acid sequences of *Cellulose synthase*s in *P. equestris*. Conserved cysteine residues are marked by red frames.

Supplementary Figure S7 Multiple alignment of the deduced amino acid sequences of *Cellulose synthase* in *A. shenzhenica*. Conserved cysteine residues are marked by red frames.

Supplementary Figure S8 Sequence structures of the alternative splicing transcripts of the *D. officinale* genes. UTRs and exons are indicated using blue and yellow rectangles, respectively. The solid lines indicate introns. The scale bar (bp) indicates the length of the corresponding transcripts.
