# Supplementary figures and images for: Species-Specific Gene Expansion of the Cellulose synthase Gene Superfamily in the Orchidaceae Family and Functional Divergence of Mannan Synthesis-Related Genes in Dendrobium officinale
Source: Front Plant Sci. 2022 Jun 3;13:777332. doi: 10.3389/fpls.2022.777332 (PMC9204230; doi:10.3389/fpls.2022.777332)

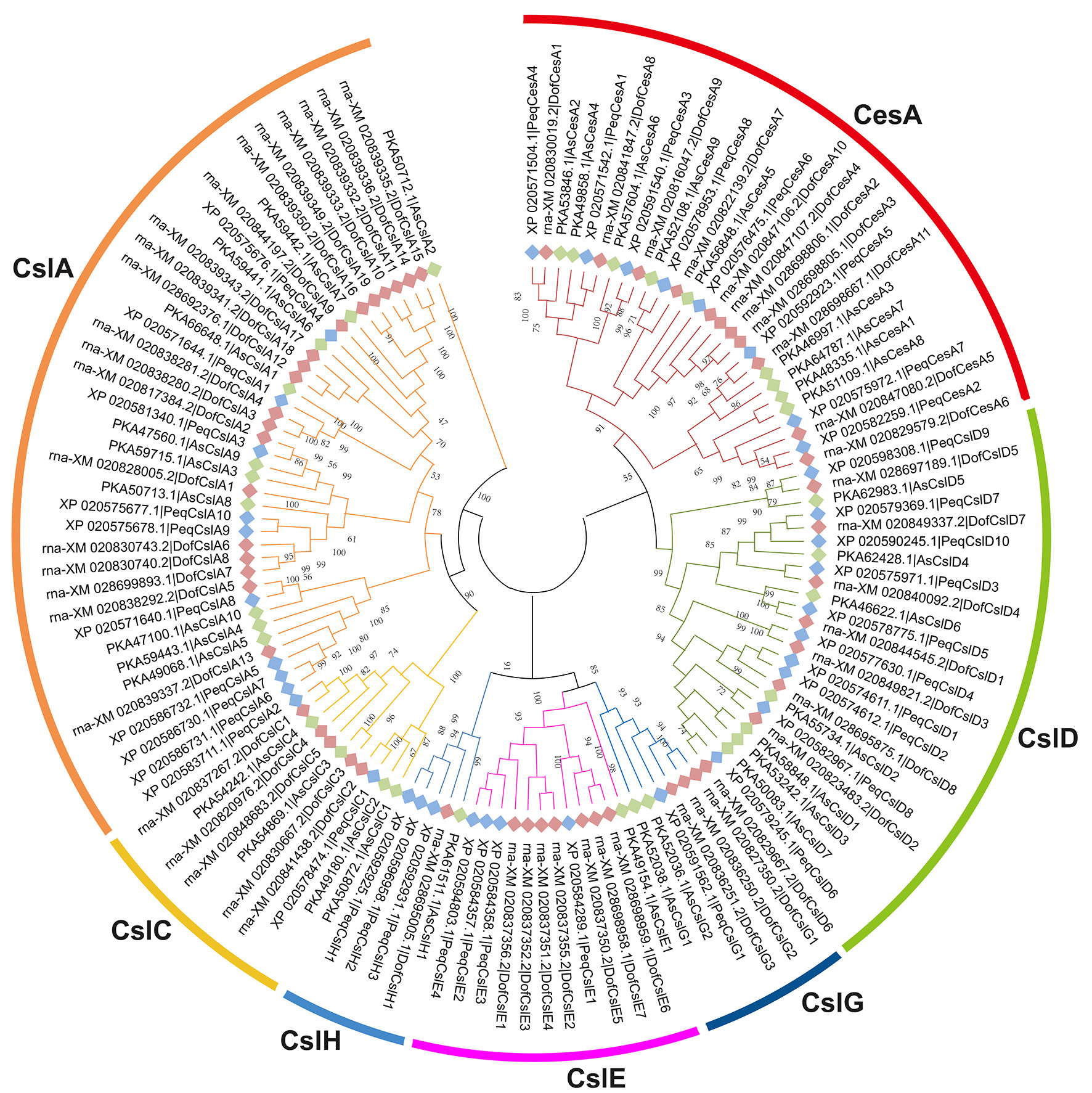

Supplement: Supplementary Figure S1 — Phylogenetic tree of the Cellulose synthase superfamily members in D. officinale (Dof), A. shenzhenica (Apo), and P. equestris (Peq). The phylogenetic tree was constructed using MEGA 6.0 with the neighbor-joining (NJ) method and 1000 bootstrap replicates. The CesA/Csl proteins were grouped into one CesA family and six Csl families: CslA, CslC, CslD, CslE, CslG, and CslH. The families are marked by different arc lines and branch colors. The D. officinale, A. shenzhenica, and P. equestris are distinguished by red, green and blue rhombuses, respectively. [file Image_1.JPEG]

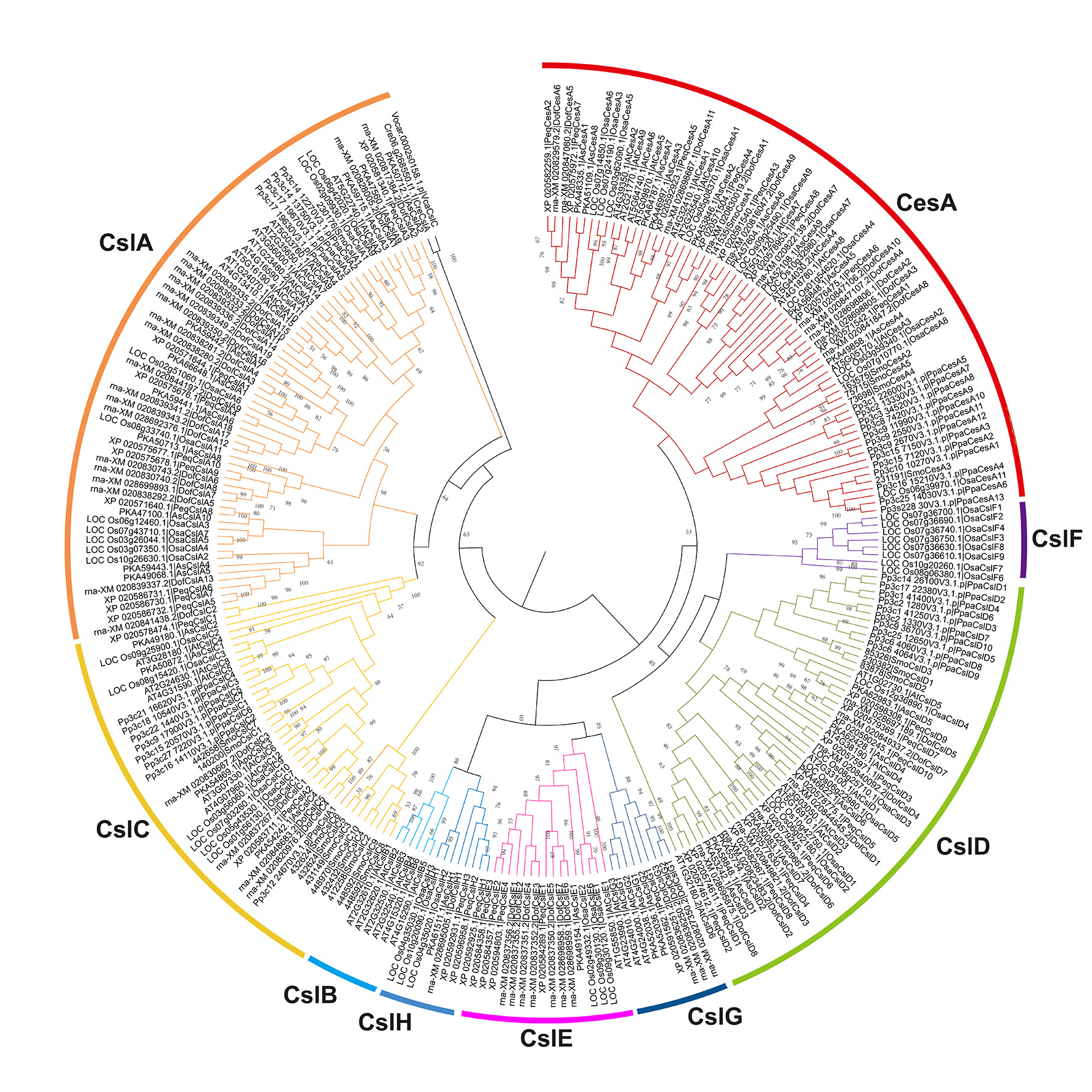

Supplement: Supplementary Figure S2 — Phylogenetic tree of Cellulose synthase superfamily members from nine representative plant species. The phylogenetic tree was constructed using MEGA 6.0 with the neighbor-joining (NJ) method and 1000 bootstrap replicates. The CesA/Csl proteins were grouped into nine families: CesA, CslA, CslB, CslC, CslD, CslE, CslF, CslG, and CslH. The species included (with the gene code prefixes shown in parentheses) are as follows: Chlamydomonas reinhardtii (Cre), Volvox carteri (Vca), Physcomitrella patens (Ppa), Selaginella moellendorffii (Smo), A. shenzhenica (Apo), D. officinale (Den), P. equestris (Peq), Oryza sativa (Osa), and Arabidopsis thaliana (AT). The families are marked by different arc lines and branch colors. [file Image_2.JPEG]

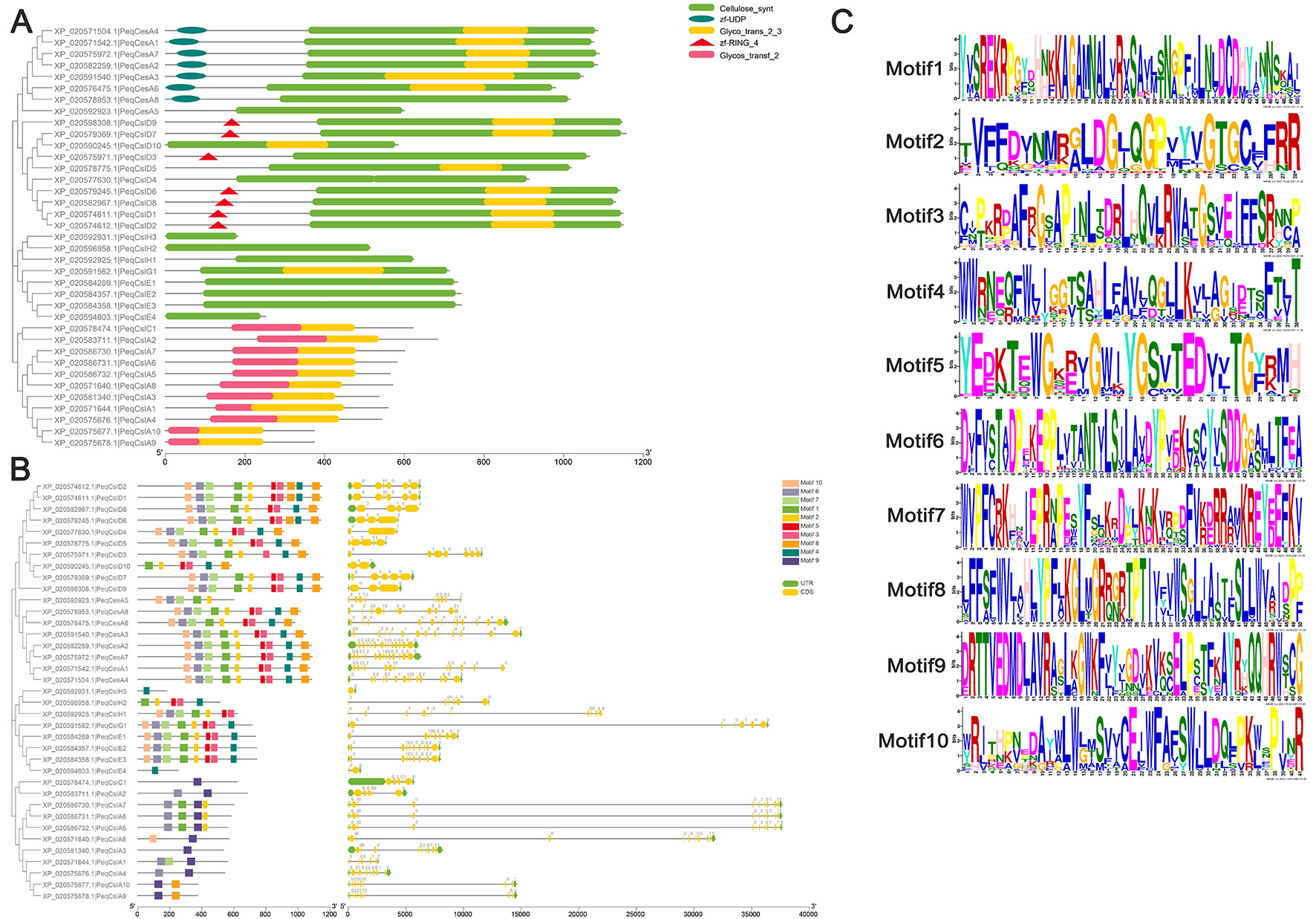

Supplement: Supplementary Figure S3 — Phylogenetic, conserved domain and motif and gene structure analyses of Cellulose synthase proteins in P. equestris (Peq). (A) Phylogenetic relationships and conserved domains of the PeqCesA/PeqCsl proteins. The scale bar (aa) indicates the amino acid position in the corresponding conserved domain. (B) Phylogenetic relationships, conserved motifs and gene structure of PeqCesA/PeqCsl genes. UTRs and exons are indicated using green and yellow rectangles, respectively. The solid lines indicate introns. The numbers above the solid lines represent the intron phase. Scale bars (bp/aa) indicate the length/amino acid position of corresponding genes/proteins; (C) Sequence logos of the 10 motifs. [file Image_3.JPEG]

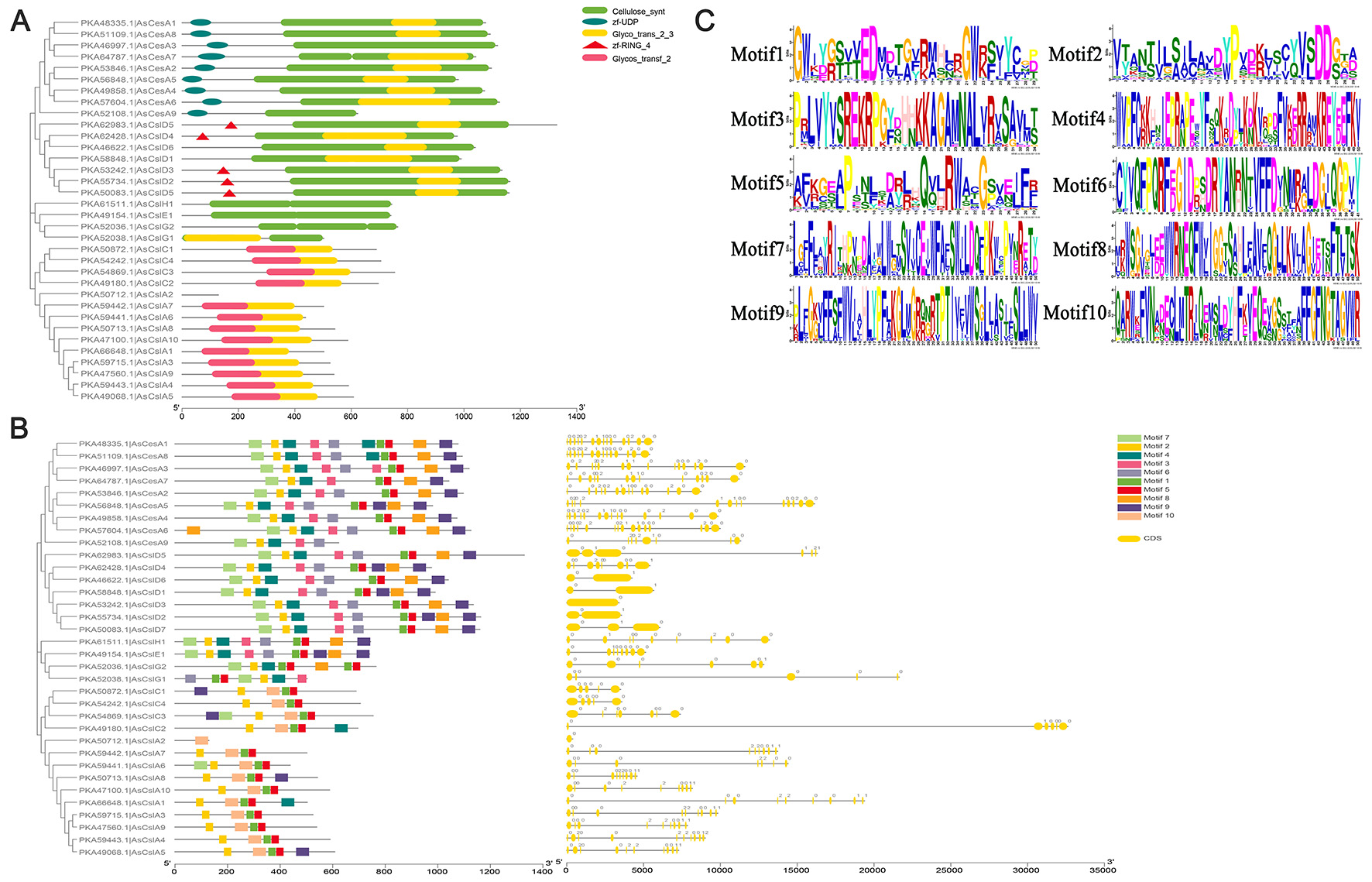

Supplement: Supplementary Figure S4 — Phylogenetic, conserved domain and motif and gene structure analyses of Cellulose synthase proteins in A. shenzhenica (Apo). (A) Phylogenetic relationships and conserved domains of the AsCesA/AsCsl proteins. The scale bar (aa) indicates the amino acid position in the corresponding conserved domains. (B) Phylogenetic relationships, conserved motifs and gene structure of the AsCesA/AsCsl genes. Exons are indicated in yellow rectangles. The solid lines indicate introns. The numbers above the solid lines represent the intron phase. The scale bars (bp/aa) indicate the length/amino acid position of corresponding genes/proteins; (C) sequence logos of the 10 motifs. [file Image_4.JPEG]

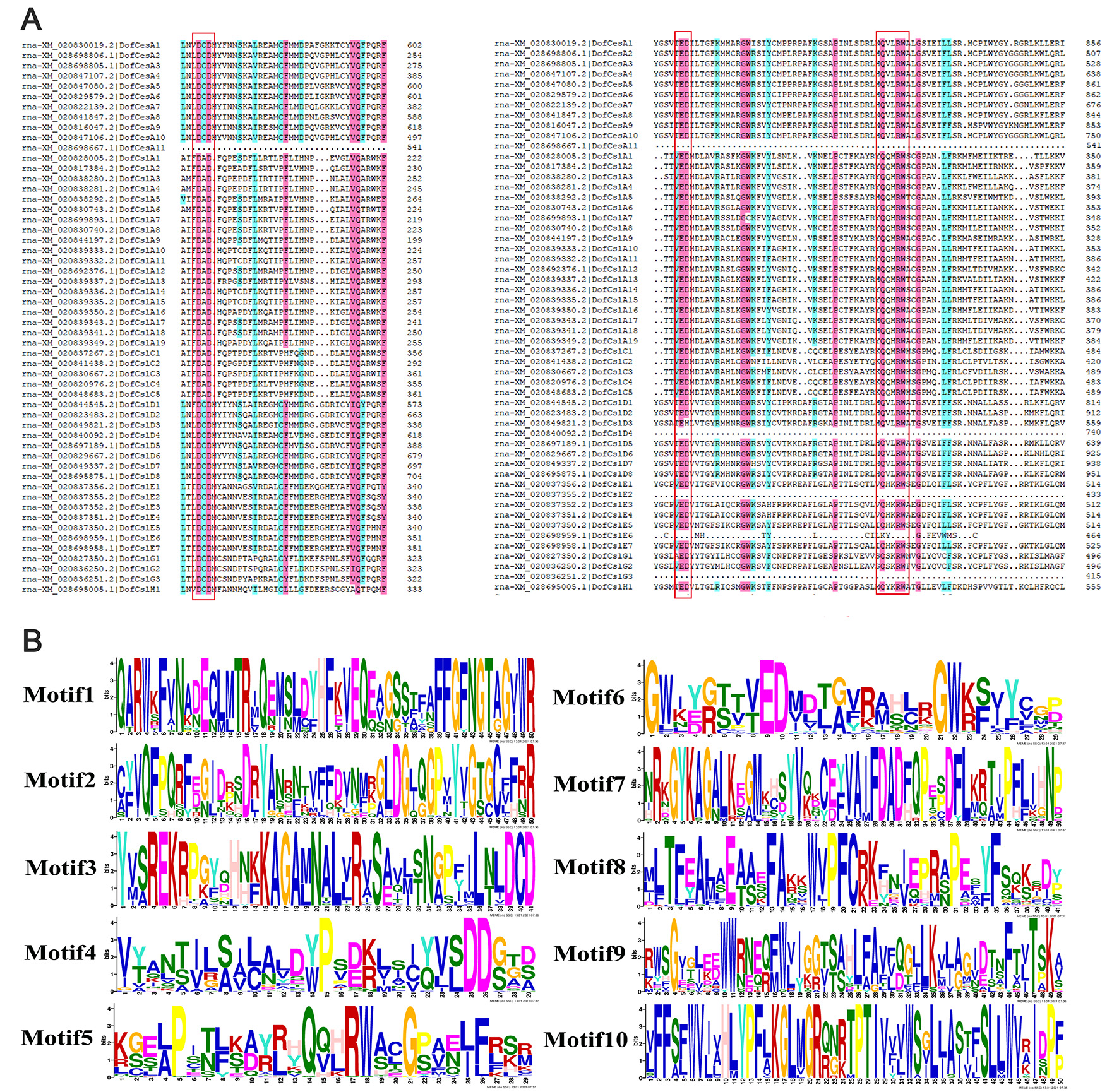

Supplement: Supplementary Figure S5 — Multiple alignment and motif logos of D. officinale. (A) Multiple comparisons of the deduced amino acid sequences of Cellulose synthase in D. officinale. Conserved cysteine residues are marked by red frames. (B) Sequence logos of the 10 motifs. [file Image_5.JPEG]

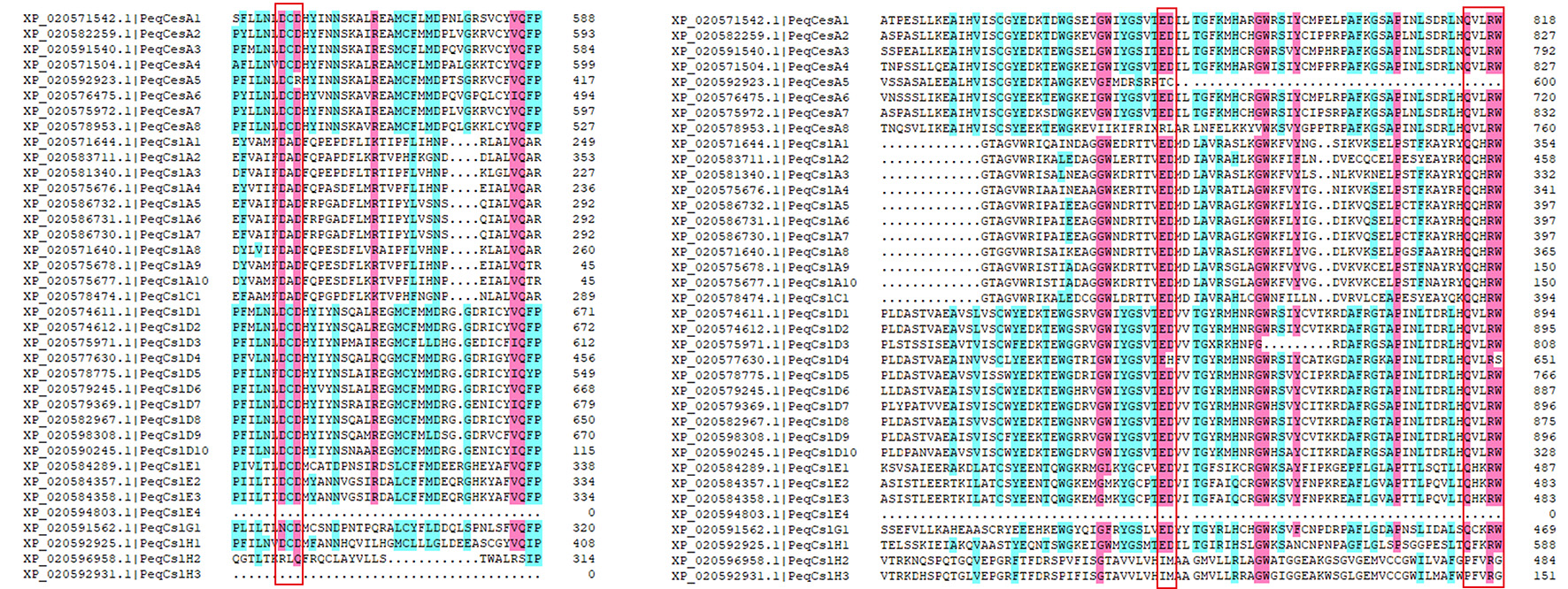

Supplement: Supplementary Figure S6 — Multiple alignment of the deduced amino acid sequences of Cellulose synthases in P. equestris. Conserved cysteine residues are marked by red frames. [file Image_6.JPEG]

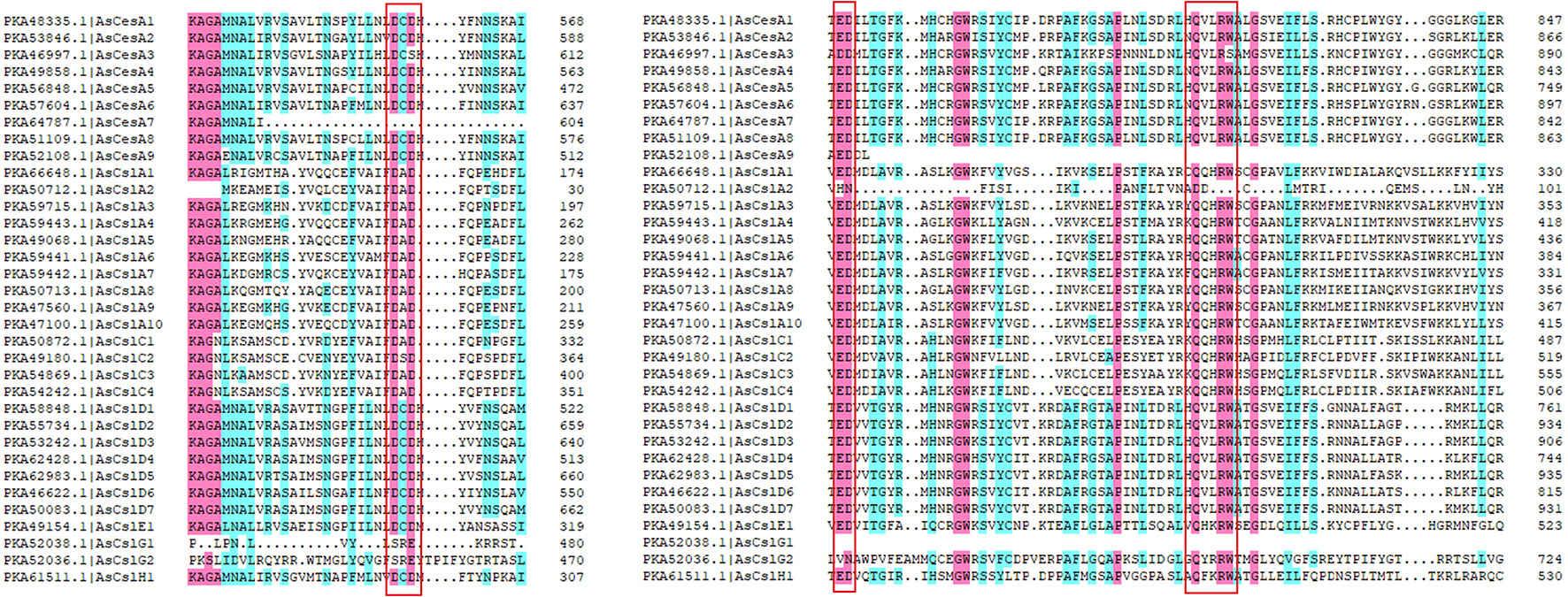

Supplement: Supplementary Figure S7 — Multiple alignment of the deduced amino acid sequences of Cellulose synthase in A. shenzhenica. Conserved cysteine residues are marked by red frames. [file Image_7.JPEG]

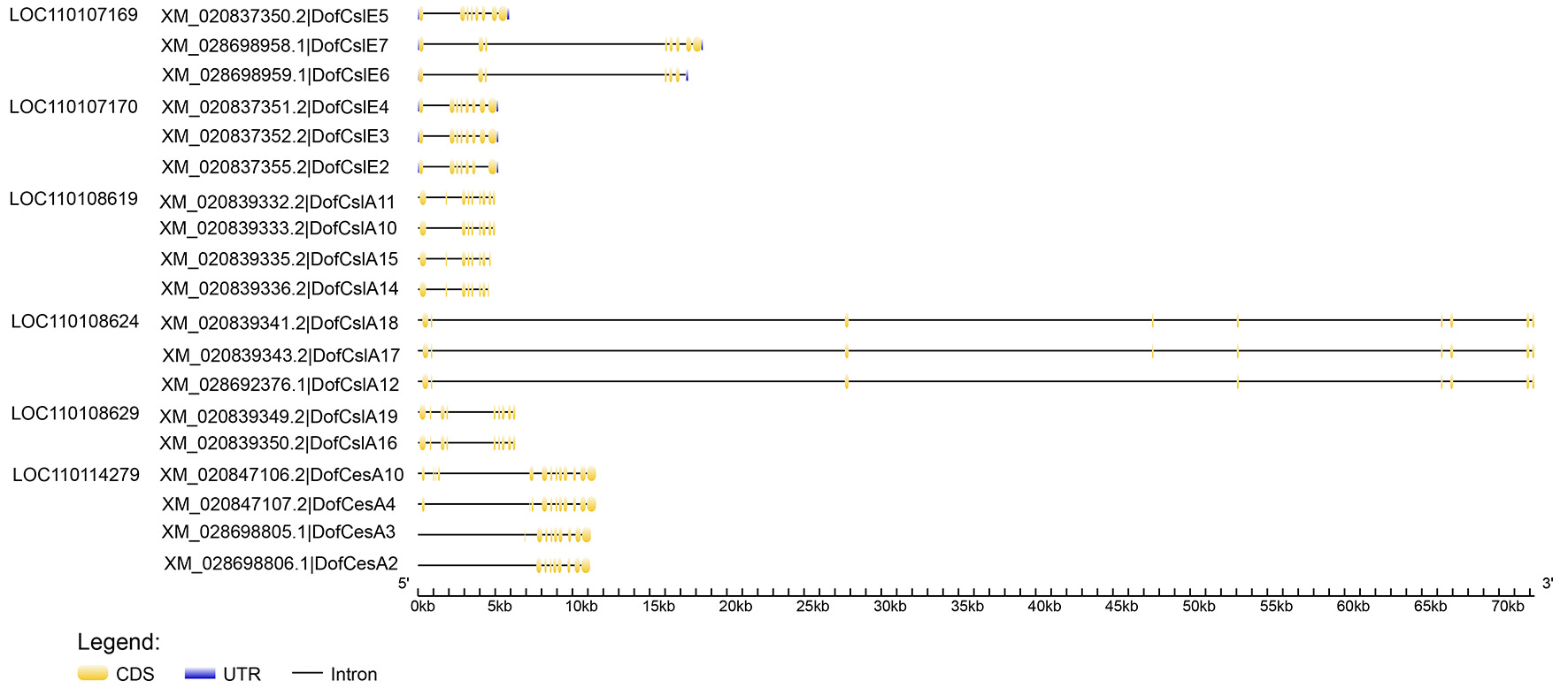

Supplement: Supplementary Figure S8 — Sequence structures of the alternative splicing transcripts of the D. officinale genes. UTRs and exons are indicated using blue and yellow rectangles, respectively. The solid lines indicate introns. The scale bar (bp) indicates the length of the corresponding transcripts. [file Image_8.JPEG]
